# Hierarchical differentiation competence in response to retinoic acid ensures stem cell maintenance during mouse spermatogenesis

**DOI:** 10.1242/dev.118695

**Published:** 2015-05-01

**Authors:** Kanako Ikami, Moe Tokue, Ryo Sugimoto, Chiyo Noda, Satoru Kobayashi, Kenshiro Hara, Shosei Yoshida

**Affiliations:** 1 Division of Germ Cell Biology, National Institute for Basic Biology, National Institutes of Natural Sciences, 5-1 Higashiyama, Myodaiji, Okazaki 444-8787, Japan; 2 Department of Basic Biology, School of Life Science, Graduate University for Advanced Studies (Sokendai), 5-1 Higashiyama, Myodaiji, Okazaki 444-8787, Japan; 3 Division of Developmental Genetics, Okazaki Institute for Integrative Bioscience, National Institute for Basic Biology, National Institutes of Natural Sciences, 5-1 Higashiyama, Myodaiji, Okazaki 444-8787, Japan

**Keywords:** Retinoic acid receptor gamma, Spermatogenesis, Spermatogonia, Stem cell niche

## Abstract

Stem cells ensure tissue homeostasis through the production of differentiating and self-renewing progeny. In some tissues, this is achieved by the function of a definitive stem cell niche. However, the mechanisms that operate in mouse spermatogenesis are unknown because undifferentiated spermatogonia (A_undiff_) are motile and intermingle with differentiating cells in an ‘open’ niche environment of seminiferous tubules. A_undiff_ include glial cell line-derived neurotrophic factor receptor α1 (GFRα1)^+^ and neurogenin 3 (NGN3)^+^ subpopulations, both of which retain the ability to self-renew. However, whereas GFRα1^+^ cells comprise the homeostatic stem cell pool, NGN3^+^ cells show a higher probability to differentiate into KIT^+^ spermatogonia by as yet unknown mechanisms. In the present study, by combining fate analysis of pulse-labeled cells and a model of vitamin A deficiency, we demonstrate that retinoic acid (RA), which may periodically increase in concentration in the tubules during the seminiferous epithelial cycle, induced only NGN3^+^ cells to differentiate. Comparison of gene expression revealed that retinoic acid receptor γ (*Rarg*) was predominantly expressed in NGN3^+^ cells, but not in GFRα1^+^ cells, whereas the expression levels of many other RA response-related genes were similar in the two populations. Ectopic expression of RARγ was sufficient to induce GFRα1^+^ cells to directly differentiate to KIT^+^ cells without transiting the NGN3^+^ state. Therefore, RARγ plays key roles in the differentiation competence of NGN3^+^ cells. We propose a novel mechanism of stem cell fate selection in an open niche environment whereby undifferentiated cells show heterogeneous competence to differentiate in response to ubiquitously distributed differentiation-inducing signals.

## Abstract (precis)

**Highlighted article:** In the open environment of mouse seminiferous tubules, an undifferentiated stem cell pool is maintained by the differential expression of the retinoic acid receptor-γ in spermatogonia.

## INTRODUCTION

The integrity of tissue homeostasis is maintained by the balanced self-renewal and differentiation of stem cells. In certain tissues, stem cells cluster in an anatomically specialized (‘closed’ or ‘definitive’) niche that directs cell fate decisions. These include the testicular hub and ovariolar cap cells in *Drosophila*, gonadal distal tip cells of *Caenorhabditis elegans* and mammalian intestinal crypts. Because niche-derived signals appear to be spatially restricted, cells that are located within a particular region (e.g. in direct contact with niche cells) can be maintained in an undifferentiated state, and their displacement from the niche leads to differentiation. In other tissues, stem cells appear to be distributed over an extended area, designated as an ‘open’ or ‘facultative’ niche. In such tissues, the details of the mechanism that determines whether stem cells differentiate or remain undifferentiated are unknown (Fuller and Spradling, 2007; Morrison and Spradling, 2008; Stine and Matunis, 2013).

**Fig. 1.**
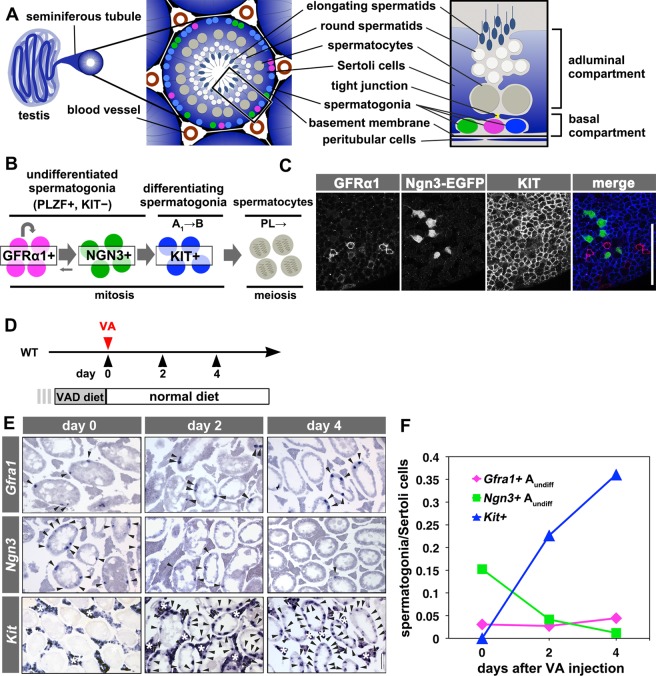
**Testis anatomy and spermatogonial populations and their kinetics in the VAD model.** (A) Anatomy of seminiferous tubules and seminiferous epithelium. A_undiff_ spermatogonia, including GFRα1^+^ (magenta) and NGN3^+^ (green) cells and KIT^+^ differentiating spermatogonia (blue), reside in the basal compartment (between the basement membrane and the tight junction of Sertoli cells). (B) Hierarchical and heterogeneous composition of spermatogonia. PL, preleptotene spermatocytes. (C) Representative whole-mount IF images of spermatogonia derived from an *Ngn3-EGFP* mouse triple stained for GFRα1, GFP and KIT. (D) The experimental schedule for E and F. Wild-type VAD mice were injected with VA and fed a normal (VA-sufficient) diet thereafter, before analysis at the indicated times. (E) Representative images of *in situ* hybridization analysis of *Gfra1*, *Ngn3* and *Kit* expression in testis sections. Arrowheads indicate spermatogonia expressing these genes. Note the persisting *Kit* expression in interstitial cells (asterisks). (F) Counts of *Gfra1*^+^, *Ngn3*^+^ and *Kit*^+^ spermatogonia. Raw counts are summarized in supplementary material Table S1. Scale bars: 100 μm.

Mouse spermatogenesis occurs in seminiferous tubules and represents a typical example of an open niche-supported stem cell system (Fig. 1A) (Russell et al., 1990; Stine and Matunis, 2013). Here, the vast majority of stem cell activity resides in a small, primitive subset of germ cells called ‘undifferentiated spermatogonia’ (A_undiff_) (Ohbo et al., 2003; Shinohara et al., 2000; Yoshida, 2012). A_undiff_ continuously give rise to ‘differentiating spermatogonia’, which include a series of cell types from A_1_ through A_2_, A_3_, A_4_, intermediate and B. Type B spermatogonia then undergo meiosis (the cells are now designated spermatocytes) and spermiogenesis. All spermatogonia (A_undiff_ through B) reside within the basal compartment of the seminiferous tubules (between the basement membrane and the junctional network of Sertoli cells); they translocate across the tight junction when they enter meiosis (Fig. 1A,B) (de Rooij and Russell, 2000). In the basal compartment, A_undiff_ localize preferentially to the area adjacent to the vasculature network of arterioles and venules that accompanies interstitial cells, including Leydig cells (Chiarini-Garcia et al., 2001, 2003; Hara et al., 2014; Yoshida et al., 2007). In this area, however, A_undiff_ do not cluster to a restricted domain but intermingle with differentiating spermatogonia (Fig. 1C). Moreover, live imaging studies demonstrate the prevalent migration of A_undiff_ between differentiating spermatogonia and immotile Sertoli cells (Hara et al., 2014; Klein et al., 2010; Yoshida et al., 2007). Therefore, it is unlikely that the microenvironment of A_undiff_ is unique compared with that of the differentiating spermatogonia.

A_undiff_ spermatogonia in the basal compartment are exposed to extracellular signals that control their self-renewal and differentiation. Maintenance of A_undiff_ *in vivo* depends on the function of glial cell line-derived neurotrophic factor (GDNF) expressed by Sertoli cells (Meng et al., 2000; Yomogida et al., 2003). *In vitro*, GDNF is required for the maintenance of spermatogonia that retain post-transplantation colony-forming stem cell activity (Kanatsu-Shinohara et al., 2003; Kubota et al., 2004). By contrast, differentiation of A_undiff_ to A_1_ spermatogonia depends on retinoic acid (RA), an active metabolite of vitamin A (VA or retinol), the synthesis and inactivation of which are mediated by enzymes expressed by Sertoli and differentiating germ cells (Sugimoto et al., 2012; Vernet et al., 2006b). In vitamin A deficiency (VAD), insufficient RA is produced and spermatogenesis is affected primarily by inhibition of the differentiation of A_undiff_ to A_1_. Administration of exogenous VA to VAD mice restarts this process of differentiation (Morales and Griswold, 1987; Sugimoto et al., 2012). A similar, but weaker, spermatogenesis defect was observed in mice lacking functional RA receptor (RAR) genes (*Rarg* and/or *Rara*) in spermatogonia, indicating that spermatogonia are (at least in part) the direct target of RA and that these receptors are involved in this differentiation process (Gely-Pernot et al., 2012). These findings warrant determining the precise target cell type(s) of RA and the roles of RA in their differentiation.

In seminiferous tubules, spermatogenesis proceeds in a periodic manner termed the ‘seminiferous epithelial cycle’ (Leblond and Clermont, 1952; Oakberg, 1956). The 8.6-day cycle of mice is divided into stages I to XII (Russell et al., 1990). The expression of enzymes that mediate RA metabolism is synchronized to the cycle, suggesting that the RA concentration increases around stages VII to VIII (Hasegawa and Saga, 2012; Sugimoto et al., 2012; Vernet et al., 2006b). Very recently, the measurement of RA levels in seminiferous epithelial cycle-synchronized mouse testis provided data supporting this contention, although this remains to be experimentally demonstrated under physiological conditions (Hogarth et al., 2015). Consistent with this, the differentiation of A_undiff_ to A_1_ occurs specifically during stages VIII to IX and accompanies the induction of a germ cell-specific RA responsive gene, *Stra8* (Mark et al., 2008; Zhou et al., 2008). However, a significant number of A_undiff_ remain undifferentiated during these stages, and A_undiff_ spermatogonia are present throughout the cycle (de Rooij and Russell, 2000; Huckins and Oakberg, 1978; Tagelenbosch and de Rooij, 1993). If all A_undiff_ are uniformly exposed to RA, which is a strong inducer of differentiation, this raises an important question about the mechanism that ensures the preservation of undifferentiated cells while producing differentiating cells.

A_undiff_ comprise singly isolated spermatogonia (A_single_ or A_s_) and interconnected syncytia of two (A_paired_ or A_pr_) or more (mainly 4, 8 and 16) cells (A_aligned_ or A_al_) (de Rooij and Russell, 2000; Huckins and Oakberg, 1978; Oakberg, 1971). In this population, gene expression profiles are heterogeneous (Hofmann et al., 2005; Meng et al., 2000; Nakagawa et al., 2010; Raverot et al., 2005; Sada et al., 2009; Suzuki et al., 2012, 2009; Yoshida et al., 2004; Zheng et al., 2009). In particular, GFRα1, a component of the GDNF receptor, and neurogenin 3 (NGN3, or NEUROG3), a basic helix-loop-helix transcription factor, are reciprocally expressed in A_undiff_; GFRα1^+^ cells mainly comprise A_s_ and A_pr_, whereas NGN3^+^ cells (most of which are GFRα1^–^) include a majority of A_al_ and a smaller number of A_pr_ and A_s_ (Hofmann et al., 2005; Nakagawa et al., 2010; Yoshida, 2012; Yoshida et al., 2004).

In homeostatic spermatogenesis, pulse-labeling studies using inducible Cre recombinase demonstrate that the GFRα1^+^ population comprises the actual stem cell pool, while producing NGN3^+^ progeny (Hara et al., 2014; Sada et al., 2009), and that most NGN3^+^ cells transit to KIT^+^ type A_1_ spermatogonia that eventually mature into spermatozoa (Fig. 1B) (Nakagawa et al., 2007, 2010; Yoshida et al., 2004). However, NGN3^+^ cells are not irreversibly committed but retain the potential to revert to GFRα1^+^ and contribute to long-term self-renewal. This process of reversion becomes prominent when tissues are damaged or when these cells are transplanted into germ cell-depleted seminiferous tubules of the host (Nakagawa et al., 2007, 2010). Therefore, the stem cell phenotype and that of differentiating cells coexist in NGN3^+^ spermatogonia, which are referred to as ‘potential stem cells’ (Nakagawa et al., 2007, 2010; Potten and Loeffler, 1990). By changing their behavior according to tissue context, NGN3^+^ spermatogonia make a significant contribution to maintaining tissue homeostasis (Nakagawa et al., 2007). Cells with similar flexibility have been identified in other stem cell systems, including *Drosophila* oogenesis and spermatogenesis, and the mammalian intestinal epithelium (Brawley and Matunis, 2004; Kai and Spradling, 2004; van Es et al., 2012), suggesting that a common mechanism maintains tissue homeostasis. However, the molecular mechanisms that impart NGN3^+^ cells with the property of potential stem cells are unknown.

In the present study, we challenged the mechanisms of fate selection of A_undiff_ to differentiate or to remain undifferentiated in the open niche environment of seminiferous tubules. We found that GFRα1^+^ and NGN3^+^ subpopulations of A_undiff_ show a differential competence to differentiate in response to RA, which is achieved by restricted RARγ expression in NGN3^+^ cells. We propose a novel mode of fate selection of stem cells whereby the heterogeneous competence to differentiate in the pool of undifferentiated cells plays important roles in directing them to differentiate or to remain undifferentiated in an open niche environment in which differentiation-inducing signals are ubiquitously distributed. As a mechanism analogous to the asymmetric divisions in some closed niche-supported systems, such heterogeneous differentiation competence would be paradigmatic for open niche-supported tissues.

## RESULTS

### Kinetics of GFRα1^+^ and NGN3^+^ spermatogonia in the VAD/VA readministration model

To investigate the response of the GFRα1^+^ and NGN3^+^ subsets of A_undiff_ to changes in tissue RA levels, we used the VAD model in which RA production is severely affected. In the testes of VAD mice, the generation of KIT^+^ spermatogonia from A_undiff_ is blocked, leaving only KIT^−^ A_undiff_. When VA (in particular, retinol, which is converted to RA) is administered, the genesis of KIT^+^ cells is reinitiated, followed by normal spermatogenesis (Morales and Griswold, 1987; Sugimoto et al., 2012; van Pelt and de Rooij, 1990).

Using this established model, we first determined the kinetics of *Gfra1*^+^, *Ngn3*^+^ and *Kit*^+^ spermatogonia by *in situ* hybridization (Fig. 1D-F). In VAD testes, although *Kit^+^* spermatogonia were lost, both *Gfra1*^+^ and *Ngn3*^+^ cells were observed (Fig. 1E, day 0). When VA was replaced, *Kit*^+^ spermatogonia appeared and their number increased rapidly, whereas the number of *Ngn3*^+^ cells decreased greatly over 4 days. However, the number of *Gfra1*^+^ cells did not change (Fig. 1E,F; supplementary material Table S1). Given that these data, as obtained from static analysis, reflect the dynamic behaviors of spermatogonial subpopulations, we then analyzed the fate of A_undiff_ subsets by performing lineage-tracing experiments.

### NGN3^+^ spermatogonia differentiate efficiently into KIT^+^ cells in response to VA administration

**Fig. 2.**
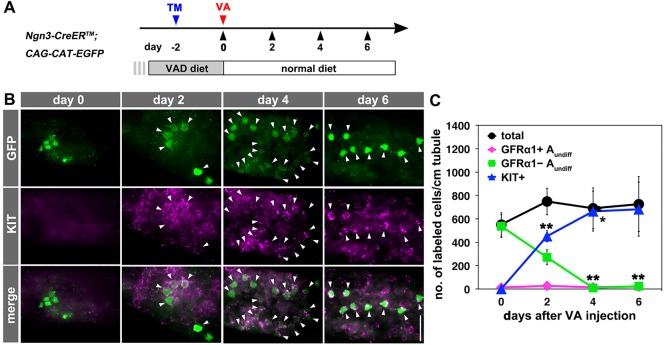
**Fate of pulse-labeled NGN3^+^ spermatogonia following administration of VA.** (A) Experimental design. Two days after the TM pulse, *Ngn3-CreER^TM^; CAG-CAT-EGFP* transgenic mice maintained in VAD were injected with VA to resume spermatogenesis. Testis samples were harvested at the indicated times. (B) IF analysis of GFP and KIT expression in whole-mount seminiferous tubules 0, 2, 4 and 6 days after VA injection. Arrowheads indicate GFP^+^/KIT^+^ double-positive cells. Note that KIT immunostaining exhibits a punctate pattern at these early stages. (C) The number of GFP-labeled GFRα1^+^ A_undiff_ (magenta), GFRα1^−^ A_undiff_ (green), KIT^+^ (blue) and total (black) cells in the testes of *Ngn3-CreER^TM^; CAG-CAT-EGFP* mice. Shown is the mean±s.e.m. of three, three, five and five testes on days 0, 2, 4 and 6, respectively. *P=0.041, **P<0.002 (*t*-test) compared with the values for day 0. Scale bar: 50 μm.

We first pulse-labeled the NGN3^+^ cells and traced their fates. NGN3^+^ spermatogonia were irreversibly labeled with green fluorescent protein (GFP) after a single pulse of 4-hydroxytamoxifen (TM) was administered to *Ngn3-CreER^TM^; CAG-CAT-EGFP* transgenic mice maintained under conditions of VAD. Following administration of VA, gene expression by the GFP-labeled cells was analyzed using whole-mount immunofluorescence (Fig. 2A). In VAD (day 0), almost all the labeled cells were negative for KIT and GFRα1 expression, as expected. After injecting VA, almost all the GFP-labeled cells expressed KIT within 4 days (Fig. 2B,C), whereas they remained GFRα1^−^ throughout (Fig. 2C; supplementary material Fig. S1A,B). These data indicate that NGN3^+^ cells transit to KIT^+^ cells rapidly and efficiently in response to RA.

### GFRα1^+^ spermatogonia do not differentiate into KIT^+^ cells in response to VA readministration

**Fig. 3.**
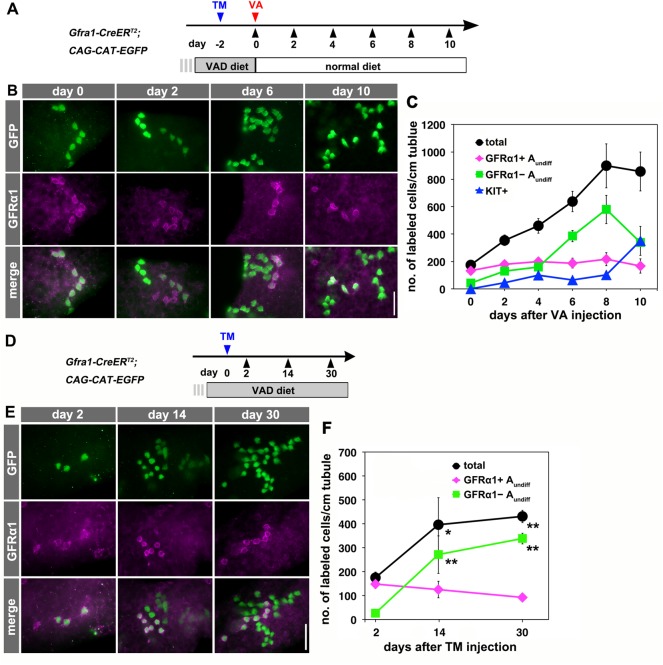
**Fate of pulse-labeled GFRα1^+^ spermatogonia during VAD/VA administration.** (A) The experimental schedule for B and C. Two days after the TM pulse, *Gfra1-CreER^T2^; CAG-CAT-EGFP* transgenic mice maintained in VAD were injected with VA and then fed a normal diet. Testis samples were harvested at the indicated times. (B) Representative IF images of whole-mount seminiferous tubule 0, 2, 6 and 10 days after VA injection stained for GFP and GFRα1. (C) The number of GFP-labeled GFRα1^+^ A_undiff_ (magenta), GFRα1^−^ A_undiff_ (green), KIT^+^ (blue) spermatogonia and total labeled (black) cells. Shown is the mean±s.e.m. of five, five, seven, six, four and three testes for days 0, 2, 4, 6, 8 and 10, respectively. Data, except for those of GFRα1^+^ cells, on days 2, 6, 8 and 10 were significantly different compared with the values on day 0 (*P*<0.03, *t*-test). (D) Schedule for E and F. After the TM pulse, *Gfra1-CreER^T2^; CAG-CAT-EGFP* transgenic mice were continually fed the VAD diet. (E) Representative IF images of whole-mount seminiferous tubule 2, 14 and 30 days after TM pulse stained for GFP and GFRα1. (F) Number of GFP-labeled GFRα1^+^ A_undiff_ (magenta) and GFRα1^−^ A_undiff_ (green) spermatogonia and total labeled cells (black), shown as the mean±s.e.m. of eight, three and five testes on days 0, 14 and 30, respectively. *P=0.032, **P<0.002 (*t*-test) compared with the values for day 2. Scale bars: 50 μm.

Next, we determined the fate of GFRα1^+^ spermatogonia in the same VAD model, using *Gfra1-CreER^T2^; CAG-CAT-EGFP* mice (Fig. 3A). Two days after pulse under VAD, the majority of the labeled cells were GFRα1^+^, as expected. The response to VA differed to that of NGN3^+^ cells in that the cells proliferated and the total number of labeled cells, which included a constant number of GFRα1^+^ cells, increased (Fig. 3B,C; supplementary material Fig. S2A,B). Simultaneously, the number of labeled cells that were KIT^−^/GFRα1^−^ (which largely represented NGN3^+^ cells) increased slowly until day 8, indicating that GFRα1^+^ cells generated NGN3^+^ cells while maintaining the size of the GFRα1^+^ population. Between days 8 and 10 after VA administration, the number of these labeled KIT^−^/GFRα1^−^ cells decreased significantly, whereas the number of labeled KIT^+^ cells increased (Fig. 3C). This reflects the second round of NGN3^+^ to KIT^+^ differentiation, which occurs when the seminiferous epithelium returns to stages VIII to IX after one cycle of 8.6 days (Sugimoto et al., 2012).

We detected a small number of labeled KIT^+^ cells prior to day 8. However, these cells might have been derived from the small number of NGN3^+^ cells detected on day 0 (recognized as KIT^−^/GFRα1^−^ cells) by the action of RA, rather than by the direct differentiation of GFRα1^+^ to KIT^+^ cells. A parallel experiment using GFP protein as a short-term lineage tracer in *Ngn3-EGFP* mice demonstrated that virtually all of the KIT^+^ cells that appeared shortly after VA administration were positive for GFP (supplementary material Fig. S2C,D). Therefore, these cells were likely to be derived from NGN3^+^ cells (see below). Taken together, these results indicate that, in response to RA, GFRα1^+^ cells do not give rise to KIT^+^ spermatogonia directly (Fig. 3C). This raises the question of whether the GFRα1^+^ to NGN3^+^ transition is induced by RA.

### The GFRα1^+^ to NGN3^+^ transition occurs under VAD

We next traced the fate of pulse-labeled GFRα1^+^ cells in *Gfra1-CreER^T2^; CAG-CAT-EGFP* mice that were continually fed the VAD diet (Fig. 3D). Fourteen and 30 days after the pulse, GFP-labeled GFRα1^−^ cells (indicating the GFRα1^+^ cell-derived NGN3^+^ cells) appeared and increased in number (Fig. 3D-F). Moreover, the number of labeled GFRα1^+^ cells remained stable (Fig. 3E,F). Therefore, under conditions of VAD, GFRα1^+^ cells proliferated and generated NGN3^+^ cells while maintaining their population size.

These findings suggest that the appearance of a small number of labeled KIT^−^/GFRα1^−^ (largely corresponding to NGN3^+^) cells 2 days after the pulse during VAD (Fig. 3F) was caused by the GFRα1^+^ to NGN3^+^ transition during this short time. By contrast, during VAD, labeled NGN3^+^ cells were rarely found to become GFRα1^+^. Instead, the total number of labeled cells decreased, indicating that NGN3^+^ cells died in the VAD testis (supplementary material Fig. S2E-G) and were effectively replenished by NGN3^+^ cells newly generated from GFRα1^+^ cells (Fig. 3F).

Taken together, these observations indicate that, in response to RA, NGN3^+^ cells quickly and efficiently transited to KIT^+^ spermatogonia, whereas GFRα1^+^ cells did not differentiate into KIT^+^ cells directly. These results may be confirmatory of previous studies showing that RA induces the transition of A_undiff_ to A_1_ spermatogonia and that virtually all the differentiating spermatogonia are derived from NGN3^+^ A_undiff_ (Morales and Griswold, 1987; Nakagawa et al., 2010; van Pelt and de Rooij, 1990). In addition, the present study is the first to demonstrate that the generation of NGN3^+^ cells from GFRα1^+^ cells occurs in the absence of RA signaling.

### Differential RARγ expression in GFRα1^+^ and NGN3^+^ A_undiff_ subsets

We next addressed the mechanism of RA induction of NGN3^+^, but not GFRα1^+^, cell differentiation. Theoretically, GFRα1^+^ cells may reside where the strength of the RA signal is insufficient. However, the expression patterns of RA metabolism-related enzymes and of a germ cell-specific RA response gene, *Stra8*, do not support this possibility (supplementary material Fig. S3A) (Sugimoto et al., 2012; Vernet et al., 2006a). In addition, GFRα1^+^ and NGN3^+^ cells were motile and intermingled with KIT^+^ differentiating spermatogonia, further contradicting this scenario (Fig. 1C) (Hara et al., 2014; Yoshida et al., 2007). Therefore, the differential response to RA is likely to reflect their intrinsic phenotype.

**Fig. 4.**
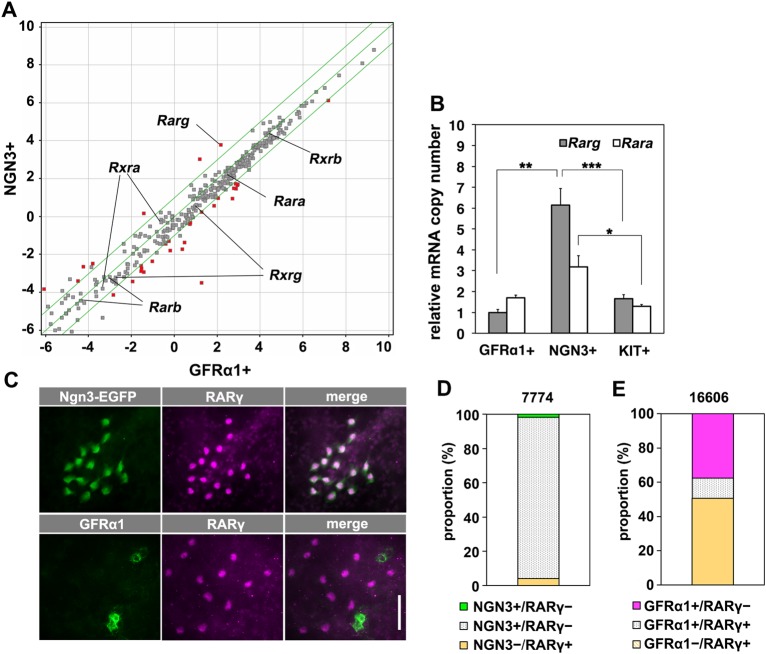
**Expression of genes in the RA signaling pathway and the specificity of RARγ expression by NGN3^+^ spermatogonia.** (A) Scatter plot comparing the levels of transcripts expressed by GFRα1^+^ and NGN3^+^ cells according to microarray analysis. For each fraction, average values from three independent RNA samples sorted from different animals are shown. The middle line indicates a difference of 0, and the outer lines represent ratios {log_2_([NGN3^+^]/[GFRα1^+^])} of 1.0 and −1.0. Red dots indicate genes expressed at significantly different levels (*P*<0.05, *t*-test with Benjamini–Hochberg correction). Members of the Rar and Rxr gene families are indicated. A list of the genes in this panel is shown in supplementary material Table S2. (B) qRT-PCR analysis of *Rara* and *Rarg* mRNA expression in GFRα1^+^, NGN3^+^ and KIT^+^ spermatogonia. Relative copy numbers are shown in arbitrary units after normalization to the amount of *Actb* mRNA. The mean±s.e.m. values of three independent cell preparations from different animals are shown. *P=0.043, **P=0.011 and ***P=0.0065 (*t*-test). (C) Representative IF images of whole-mount seminiferous tubules from *Ngn3-EGFP* mice stained for EGFP, GFRα1 and RARγ. (D,E) Frequency of single- and double-positive spermatogonia for the expression of RARγ and NGN3 (recognized as RARγ^+^/KIT^−^ and NGN3-EGFP^+^/KIT^−^, respectively) (D) and of RARγ and GFRα1 (E). Total counts are shown above each bar. Scale bar: 50 μm.

We next addressed the state of RA signaling in GFRα1^+^ and NGN3^+^ spermatogonia. Gene expression microarray analysis was performed using spermatogonia isolated from the testes of *Gfra1-EGFP* and *Ngn3-EGFP* mice, respectively, using fluorescence-activated cell sorting (FACS) (Uesaka et al., 2007; Yoshida et al., 2004). For the majority of transcripts related to the RA signaling pathway that were detected, the levels were similar in GFRα1^+^ and NGN3^+^ cells (Fig. 4A). By contrast, the differential expression of *Rarg*, which encodes one of the three RARs, was prominently upregulated in NGN3^+^ cells, as confirmed by qRT-PCR analysis (Fig. 4A,B; supplementary material Table S2).

Immunohistochemical analysis of RARγ expression demonstrated that it was predominantly expressed by spermatogonia in the testis (supplementary material Fig. S3B), as shown previously (Vernet et al., 2006b). Whole-mount immunostaining of seminiferous tubules from *Ngn3-EGFP* mice further demonstrated that virtually all NGN3^+^ cells showed highly consistent expression of RARγ in the nucleus (Fig. 4C,D). By contrast, GFRα1 and RARγ were reciprocally expressed, with a small portion of double-positive cells showing weaker signals for both proteins (Fig. 4C,E; supplementary material Fig. S3C). Virtually all (2057 of 2085, 98.7%) cells that were positive for PLZF, a marker of A_undiff_ spermatogonia (Buaas et al., 2004; Costoya et al., 2004), also expressed either GFRα1 or RARγ (supplementary material Fig. S3D). KIT^+^ spermatogonia showed less intense staining of RARγ (supplementary material Fig. S3E). RARγ immunoreactivity was detected not only in many NGN3^+^ A_al_ but also in a few NGN3^+^ A_s_ and A_pr_ spermatogonia (supplementary material Fig. S3F).

These observations are largely consistent with a previous report that RARγ is expressed in A_al_ cells that are GFRα1^–^ (Gely-Pernot et al., 2012), given that the majority of NGN3^+^ and GFRα1^+^ cells are A_al_ and A_s_/A_pr_, respectively (Nakagawa et al., 2010). The present study demonstrates quantitatively that RARγ expression is highly overlapping with that of NGN3, and that essentially all A_undiff_ spermatogonia express either GFRα1 or RARγ. The *Rara* transcript was detected in A_undiff_ at levels comparable to *Rarg*, although the level of the *Rara* transcript was similar between GFRα1^+^ and NGN3^+^ cells (Fig. 4A,B).

### Enforced RARγ expression imparts differentiation competence to GFRα1^+^ cells

In *Rarg* knockout mice, the genesis of differentiating spermatogonia is impaired, whereas A_undiff_ are restored (Gely-Pernot et al., 2012), indicating that RARγ is required for the normal progression of NGN3^+^ to KIT^+^ differentiation. However, whether other components are involved in the differentiation of NGN3^+^ cells in response to RA is unknown. Nevertheless, considering the largely similar expression levels of the other RA signaling-related genes (Fig. 4A) and the key function of RARγ in mediating the RA signal, we tested whether the induction of RARγ expression is sufficient to provide GFRα1^+^ cells with differentiation competence.

**Fig. 5.**
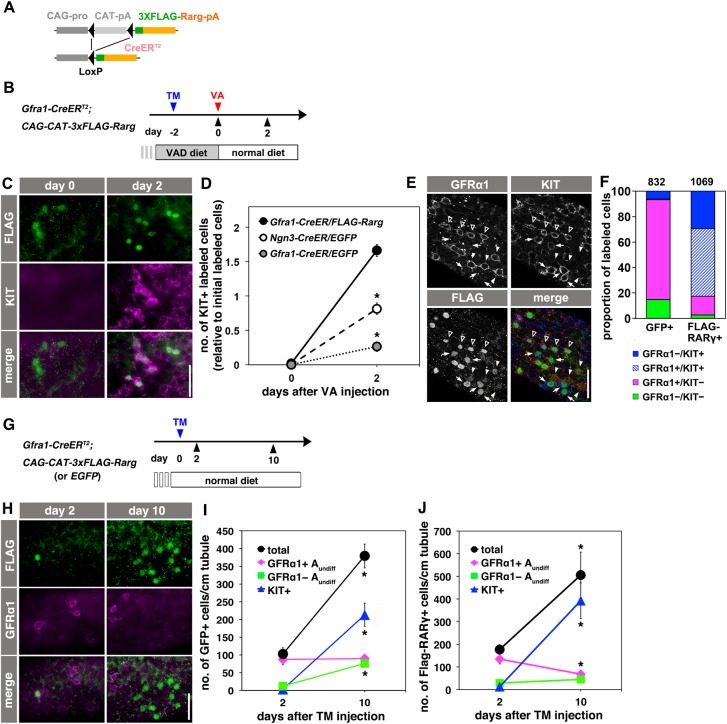
**Ectopic RARγ expression by GFRα1^+^ spermatogonia.** (A) The *CAG-CAT-3xFLAG-Rarg* transgene. When *CAT* between the loxP sites is deleted by TM-activated Cre, FLAG-tagged RARγ is constitutively expressed under the control of the *CAG* promoter. (B) Experimental design of the fate analysis of GFRα1^+^ cells with enforced FLAG-RARγ expression upon VA readministration in VAD mice, as shown in C-F. *Gfra1-CreER^T2^; CAG-CAT-3xFLAG-Rarg* transgenic mice were maintained in VAD and VA was administered 2 days after TM injection, as indicated. Testes were then processed for IF. (C,D) IF images of whole-mount seminiferous tubules of the mice described above, 2 days after VA injection, stained for FLAG-RARγ (green) and KIT (magenta) (C), and cell number relative to the number of initial induced cells (D). Data for GFP-labeled NGN3^+^ and GFRα1^+^ cells are reproduced from Fig. 2C and Fig. 3C, respectively, for comparison. The mean±s.e.m. value of three testes is shown. *P<0.003 (*t*-test), compared with the values of FLAG-RARγ^+^ GFRα1^+^ cells at day 2. (E,F) Representative confocal images of the same field of whole-mounts of seminiferous tubules of mice treated as described above, at 2 days after VA injection; staining was performed for GFRα1, KIT and FLAG (E). Open arrowheads, white arrowheads and small arrows indicate FLAG^+^ cells that are GFRα1^+^/KIT^+^, GFRα1^+^/KIT^−^ and GFRα1^−^/KIT^+^, respectively. (F) Quantitation of GFP^+^ and FLAG-RARγ^+^ cells showing different patterns of GFRα1 and KIT expression in *Gfra1-CreER^T2^; CAG-CAT-EGFP* and *Gfra1-CreER^T2^; CAG-CAT-3xFLAG-Rarg* mice, respectively*.* Cell numbers are shown above each bar. (G) Experimental design of the fate analysis of GFRα1^+^ cells with enforced FLAG-RARγ expression under normal conditions, as shown in H-J. *Gfra1-CreER^T2^; CAG-CAT-3xFLAG-Rarg* transgenic mice were pulsed with TM at 13-17 weeks of age, and after 2 and 10 days their testes were processed for IF. (H) IF images of whole-mount seminiferous tubules 2 and 10 days after TM injection, stained for FLAG-RARγ and GFRα1. (I,J) Numbers of GFRα1^+^ A_undiff_ (magenta), GFRα1^−^ A_undiff_ (green), KIT^+^ (blue) spermatogonia and total cells (black) in either GFP-labeled (I) or FLAG-RARγ-expressing (J) cells of *Gfra1-CreER^T2^; CAG-CAT-EGFP* and *Gfra1-CreER^T2^; CAG-CAT-3xFLAG-Rarg* mice, respectively, following the schedule shown in G. The mean±s.e.m. of four (I) and three (J) testes are shown. *P<0.05 (*t*-test), compared with the values on day 2. Scale bars: 50 μm.

We performed a gain-of-function experiment by ectopic expression of RARγ in GFRα1^+^ spermatogonia. We generated *Gfra1-CreER^T2^; CAG-CAT-3xFLAG-Rarg* mice, in which FLAG-tagged RARγ can be induced in GFRα1^+^ cells upon TM injection (Fig. 5A; supplementary material Fig. S4A,B). The behavior of GFRα1^+^ cells that expressed FLAG-RARγ was first tested in the VAD/VA administration model (Fig. 5B-F). In VAD (day 0), most of the FLAG-RARγ^+^ cells were GFRα1^+^ and KIT^−^, similar to the control GFP-labeled GFRα1^+^ cells (Fig. 3). However, when VA was replaced, FLAG-RARγ^+^ cells became KIT^+^ in 2 days, in stark contrast to GFP-labeled GFRα1^+^ cells, which largely remained KIT^−^ (Fig. 5C,D and Fig. 3C). These results clearly indicate that enforced RARγ expression provides GFRα1^+^ spermatogonia with the competence to differentiate into KIT^+^ spermatogonia, which is embodied when the RA signal is generated.

Following VA administration, FLAG-RARγ^+^/GFRα1^+^ cells expressed KIT as quickly or more quickly than NGN3^+^ cells (Fig. 5D). Further, a significant percentage of FLAG-RARγ^+^/KIT^+^ cells were positive for GFRα1, although GFRα1^+^/KIT^+^ spermatogonia were observed only rarely in control EGFP-expressing GFRα1^+^ cells following VAD/VA readministration (Fig. 5E,F), and GFRα1^+^/KIT^+^ cells are not found in untreated wild-type mouse testes (Nakagawa et al., 2010). Therefore, these findings support the conclusion that RARγ expression induces the direct transition of GFRα1^+^ to KIT^+^ in response to RA, although a very short NGN3^+^ transition state cannot be formally excluded.

We next analyzed the fates of GFRα1^+^ cells that expressed FLAG-RARγ under normal physiological conditions as compared with GFP-labeled controls (Fig. 5G-J). Two days after TM injection, ∼80% of GFP^+^ and FLAG-RARγ^+^ cells were GFRα1^+^ (Fig. 5H-J). Ten days following induction, one 8.6-day seminiferous epithelial cycle had been completed, and we assumed that all the labeled cells were exposed to the high concentration of RA. During this period, the control GFP^+^ cells proliferated and generated NGN3^+^ A_undiff_ (KIT^−^/GFRα1^−^ cells) as well as KIT^+^ differentiating spermatogonia, while maintaining a constant number of GFRα1^+^ cells (Fig. 5I). These findings are consistent with those of Hara et al. (2014). By contrast, the number of FLAG-RARγ^+^ cells that remained GFRα1^+^ decreased, and a greater percentage of FLAG-RARγ^+^ cells expressed KIT (Fig. 5H,J). Fifty days after induction, FLAG-RARγ^+^ cells were almost completely depleted from the GFRα1^+^ population (supplementary material Fig. S4C), in contrast to the long-term fate of GFP-labeled GFRα1^+^ cells, which persisted in the GFRα1^+^ population for at least a year (Hara et al., 2014). Thus, we conclude that enforced RARγ expression tilted the fate of GFRα1^+^ cells toward differentiation under physiological conditions.

In undisturbed testes, FLAG-RARγ^+^/KIT^+^ cells were observed predominantly around stages IX to XI, 4 days after TM injection. Considering the lag time between TM injection and protein induction (1-2 days), this suggests that FLAG-RARγ^+^ cells differentiated preferentially to KIT^+^ cells around stages VII to IX, when the NGN3^+^ to KIT^+^ transition normally occurs (supplementary material Fig. S4D). Because FLAG-RARγ^+^/GFRα1^+^ cells became KIT^+^ only in response to RA (Fig. 5B-D), this result is consistent with the hypothesis that the tissue RA concentration increases around stage VIII (Hasegawa and Saga, 2012; Hogarth and Griswold, 2010; Sugimoto et al., 2012).

### Kinetics of the GFRα1^+^ and NGN3^+^ populations during the seminiferous epithelial cycle

**Fig. 6.**
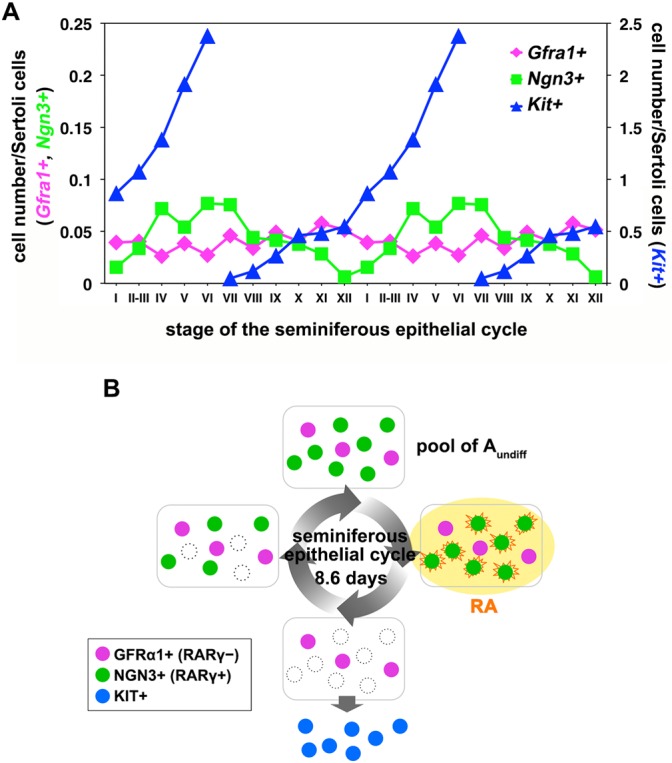
**Kinetics of GFRα1^+^, NGN3^+^ and KIT^+^ spermatogonia during the seminiferous epithelial cycle.** (A) The frequencies of *Gfra1*^+^, *Ngn3*^+^ and *Kit*^+^ spermatogonia observed during each stage of the seminiferous epithelial cycle as determined by *in situ* hybridization of three testis sections. Counts from 894 tubule transverse sections were summed and normalized to the number of Sertoli cells. Data for *Kit*^+^ spermatogonia are reproduced from Sugimoto et al. (2012). The same data are shown twice here to highlight the periodicity. (B) Model showing the behavior of GFRα1^+^ and NGN3^+^ spermatogonia during the seminiferous epithelial cycle that maintains the A_undiff_ pool while periodically producing differentiating KIT^+^ spermatogonia (see text for details).

Finally, we attempted to capture the behaviors of the GFRα1^+^ and NGN3^+^ subsets of A_undiff_, which respond differentially to RA, during the seminiferous epithelial cycle. Using *in situ* hybridization, we determined the frequency of these and of *Kit*^+^ spermatogonia (Fig. 6A; supplementary material Fig. S5). Fig. 6A presents a quantitative summary of the distributions of spermatogonial subsets as previously reported in separate studies (Grasso et al., 2012; Sugimoto et al., 2012; Yoshida et al., 2004), which reinforces morphology-based classical observations (Tagelenbosch and de Rooij, 1993). The number of *Gfra1*^+^ cells remained relatively constant throughout the cycle, as observed previously (Grasso et al., 2012). By contrast, there was a prominent increase, followed by a decrease, in the number of *Ngn3*^+^ cells during the cycle. The number of *Ngn3*^+^ cells was at its lowest in stage XII, increasing until stage VII, and then decreasing from stage VIII onwards (Fig. 6A; supplementary material Table S3). The number of *Kit*^+^ spermatogonia increased rapidly from stage VII to stage VI of the next cycle, when they transformed into preleptotene spermatocytes.

## DISCUSSION

To better understand stem cell function in tissues with an open niche environment, we investigated the mechanisms underlying the fate decision of A_undiff_ (whether to remain undifferentiated or to differentiate) during mouse spermatogenesis. Using lineage-tracing analysis combined with a VAD mouse model, we demonstrate here that NGN3^+^ and GFRα1^+^ cells show distinct competence to differentiate in response to RA: NGN3^+^ cells quickly differentiated into KIT^+^ cells in response to RA, whereas GFRα1^+^ cells remained undifferentiated. Although most of the genes related to RA signaling showed similar expression levels in the two subpopulations, *Rarg* was expressed predominantly by NGN3^+^ spermatogonia as compared with GFRα1^+^ cells. Intriguingly, enforced RARγ expression in GFRα1^+^ spermatogonia imparted these cells with the competence to differentiate in response to RA (Fig. 5).

The present study strengthens the evidence obtained by previous studies regarding the expression of RARγ and its loss-of-function phenotype during spermatogenesis (Gely-Pernot et al., 2012; Vernet et al., 2006b) and further clarifies the role of RARγ in regulating spermatogonial differentiation. Gely-Pernot et al. (2012) detected RARγ expression in some A_al_ that do not express GFRα1. These findings are supported here, leading to the conclusion that the NGN3^+^ population, including A_s_ and A_pr_ cells in addition to A_al_, express RARγ with high consistency and that essentially all A_undiff_ express either GFRα1 or RARγ with minimal overlap (Fig. 4D,E). In *Rarg* knockout mice, the transition from A_undiff_ to A_1_ was impaired, whereas A_undiff_ were restored (Gely-Pernot et al., 2012); although this prior study established the requirement of *Rarg* for this process, it remained to be determined whether RARγ is sufficient for the gain of differentiation competence. The present study demonstrates that enforced RARγ expression by GFRα1^+^ cells is sufficient for these cells to gain such competence (Fig. 5). These gain- and loss-of-function experiments establish that, upon the transition of cells from GFRα1^+^ to NGN3^+^, upregulation of *Rarg* induces the acquisition of differentiation competence.

*Rara* mRNA expression by GFRα1^+^ and NGN3^+^ spermatogonia at levels comparable to that of *Rarg* (Fig. 4A,B) predicts its proportional contribution to differentiation competence. However, a spermatogonia-specific *Rara* mutation alone does not cause any apparent defect in differentiation, while enhancing the phenotype of *Rarg* mutation (Gely-Pernot et al., 2012), indicating the auxiliary role of RARα. This might reflect the protein expression level, since RARα is expressed at undetectable levels by spermatogonia (Vernet et al., 2006b). Further, this might reflect the higher half-maximal effective concentration (EC_50_) of RARα (∼20-200 nM) compared with that of RARγ (∼0.5-2 nM) (Astrom et al., 1990; Brand et al., 1988; Giguere et al., 1990), given the reported testicular RA concentration of ∼10-20 nM (Kane et al., 2005; Obrochta et al., 2014). The local RA concentration in seminiferous tubules and its fluctuation during the cycle warrant future direct measurement.

NGN3^+^ cells represent a characteristic subpopulation of A_undiff_ that possess the potential to self-renew but are primed for differentiation, and therefore they are designated ‘potential stem cells’ (Nakagawa et al., 2007, 2010; Potten and Loeffler, 1990). The molecular basis of this hybrid property is an interesting question. The self-renewal potential might be related to their epigenomic state, since the level of repressive histone modifications (dimethylated Lys9 of histone H3) and the expression of DNA methyltransferases (Dnmt3a2 and Dnmt3b) are low in GFRα1^+^ and NGN3^+^ cells but increase dramatically in KIT^+^ cells (Shirakawa et al., 2013). By contrast, the factor(s) that primes NGN3^+^ cells for differentiation is unknown. We demonstrate here that RARγ is the key molecule for NGN3^+^ spermatogonia to gain the competence to respond to RA and differentiate. The data also indicate that GFRα1^+^ cells already express other essential RA signaling molecules and that RA target genes are already prepared for activation by RARγ in GFRα1^+^ cells.

RARγ is a potent transcriptional regulator that is active in the presence of RA. In the absence of RA, therefore, RARγ expression would not affect the cellular phenotype. These characteristics of RARγ are consistent with the ‘differentiation-primed but undifferentiated’ nature of NGN3^+^ cells. NGN3^+^ cells might be more appropriately considered as stem cells that have gained differentiation competence, rather than as transit-amplifying cells that possess de-differentiation ability. This might provide insights that will facilitate the characterization of similar cell types present in other stem cell systems.

Under physiological conditions, a fraction of GFRα1^+^ cells with ectopic expression of RARγ remained undifferentiated following one full seminiferous epithelial cycle after the induction of RARγ, although they eventually left the pool of GFRα1^+^ cells (Fig. 5J; supplementary material Fig. S4C). This might indicate the requirement for other conditions in order to embody differentiation competence in response to the intrinsic level of the RA signal. This possibility, which might involve the expression of other genes or the state of the RA target genes, warrants future investigation.

In seminiferous tubules, a strong differentiation-inducing signal (RA) appears to be abundant around stage VIII. This is consistently supported by evidence concerning the expression of enzymes required for RA metabolism, endogenous target genes and RA-responsive reporter genes (Hasegawa and Saga, 2012; Snyder et al., 2011; Sugimoto et al., 2012). The recent measurement of RA concentration in the stage-synchronized testis further strengthens this hypothesis (Hogarth et al., 2015). The present study contributes supporting evidence in that FLAG-RARγ-expressing GFRα1^+^ cells preferentially differentiated around these stages (supplementary material Fig. S4D).

Our findings suggest that heterogeneous differentiation competence in response to RA among A_undiff_ is established by the differential expression of RARγ and is essential for continual and constant sperm production. A summary of the cyclic kinetics *in vivo* is depicted in Fig. 6B. Before the RA level is increased, the entire A_undiff_ population exists as a mixture of GFRα1^+^ (RARγ^−^) and NGN3^+^ (RARγ^+^) cells around stages IV to VI (Fig. 6B, top). In stages VII to VIII, the tissue RA concentration increases and GFRα1^+^ and NGN3^+^ spermatogonia are equally exposed to RA (Fig. 6B, right). However, only NGN3^+^ (RARγ^+^) cells respond to RA and differentiate into KIT^+^ A_1_ spermatogonia, while GFRα1^+^ (RARγ^−^) cells remain undifferentiated (Fig. 6B, bottom). NGN3^+^ cells are then replenished by GFRα1^+^ cells through unknown mechanisms in the absence of RA signaling (Fig. 3D-F). Then, A_undiff_ regain heterogeneity (Fig. 6B, left), until swept away by RA in stages VII to VIII of the next cycle. Throughout the cycle, GFRα1^+^ cells continuously generate a largely consistent number of their own population regardless of the RA level. The mechanisms that control the maintenance of the GFRα1^+^ population warrant future investigation.

Although such a highly synchronized cycle of differentiation is characteristic of mouse spermatogenesis, the stem cell strategy revealed here (the genesis of a heterogeneous population of undifferentiated cells differing in their competence to differentiate, combined with ubiquitous exposure to a differentiation-inducing signal) might control other stem cell systems supported by open or facultative niches.

## MATERIALS AND METHODS

### Animals

Transgenic mice were as follows: *Ngn3-CreER^TM^* (Yoshida et al., 2006), *Gfra1-CreER^T2^* (Hara et al., 2014), *Ngn3-EGFP* (Yoshida et al., 2004), *Gfra1-EGFP* (Uesaka et al., 2007) and *CAG-CAT-EGFP* (Kawamoto et al., 2000). *CAG-CAT-3xFLAG-Rarg* transgenic mice were generated as described in the supplementary Materials and Methods. The background of all mice was C57BL/6 (Japan SLC, Japan CLEA). All animal experiments were conducted with the approval of The Institutional Animal Care and Use Committee of National Institutes of Natural Sciences, or as specified.

### VAD model

VAD mice were prepared as described (Sugimoto et al., 2012; van Pelt and de Rooij, 1990). Briefly, wild-type (WT), *Ngn3-EGFP*, *CAG-CAT-EGFP* and *CAG-CAT-3xFLAG-Rarg* female mice were fed a VA-deficient diet (Chubu Kagaku Shizai) for at least 4 weeks before being mated with appropriate males (WT, *Ngn3-EGFP*, *Ngn3-CreER^TM^* or *Gfra1-CreER^T2^*). The mother and male offspring received the same diet until the latter were used for experiments at the age of 10-14 weeks, when their body weights were maximal. To resume spermatogenesis, VAD mice were intraperitoneally administered 0.5 mg retinyl acetate (Sigma-Aldrich) dissolved in 25 μl ethanol and mixed with 75 μl sesame oil (Nacalai Tesque). The mice were subsequently fed a normal diet with a sufficient amount of VA.

### Pulse labeling and induction of FLAG-RARγ by TM

Induction of Cre-mediated recombination by TM to label the GFRα1^+^ and NGN3^+^ cells with GFP or to induce FLAG-RARγ expression was performed as described previously (Hara et al., 2014; Nakagawa et al., 2010). See the supplementary Materials and Methods for details.

### *In situ* hybridization and immunofluorescence (IF) analyses

*In situ* hybridization using paraffin sections was performed according to protocols described previously (Yoshida et al., 2001). Whole-mount IF of seminiferous tubules was performed according to a published method (Nakagawa et al., 2010). Observations and measurements were performed using an Olympus BX51 upright fluorescence microscope equipped with a DP72 CCD camera or using a Leica TCS SP8 confocal system. Detailed protocols, probes and the antibodies used are described in the supplementary Materials and Methods.

### Cell sorting

GFRα1^+^ spermatogonia fractions were collected using flow cytometry from adult *Gfra1-EGFP* mice (Uesaka et al., 2007) as the GFP^+^ fraction. NGN3^+^ and KIT^+^ fractions were collected from *Ngn3-EGFP* mice (Yoshida et al., 2004) as the GFP^+^/KIT^−^ and GFP^+^/KIT^+^ fractions, respectively, after staining with a Phycoerythrin/Cyanine 5 tandem-conjugated rat anti-mouse CD117 (KIT) antibody (Southern Biotech, #1880-13).

### Microarray and quantitative (q) RT-PCR gene expression analyses

Microarray analysis of transcripts from sorted fractions of spermatogonia was performed using a SurePrint G3 Mouse GE 8x60K Microarray Kit and G2505C or G2565CA scanner (Agilent Technologies). Three samples from different animals were analyzed for each cell type. For GFRα1^+^ and KIT^+^ fractions, each sample was collected from a single individual; samples from two mice were pooled to analyze NGN3^+^ cells. Data preparation and statistical analysis were performed using Gene Spring v12.0.0.0 (Silicon Genetics). The full microarray dataset will be published elsewhere and further details are available from the authors upon request.

qRT-PCR was performed using a LightCycler 480 system (Roche). See the supplementary Materials and Methods for detailed protocols and conditions.

## Supplementary Material

Supplementary Material
