# Supplementary material for: Hierarchical differentiation competence in response to retinoic acid ensures stem cell maintenance during mouse spermatogenesis
Source: Development. 2015 May 1;142(9):1582–92. doi: 10.1242/dev.118695 (PMC4419276; doi:10.1242/dev.118695)
Supplement: Supplementary Material [file supp_142_9_1582__index.html]

Supplementary Material 

# Hierarchical differentiation competence in response to retinoic acid ensures stem cell maintenance during mouse spermatogenesis

## DEV118695 Supplementary Material

**Files in this Data Supplement:**

- Supplementary Material
