# Supplementary material for: Hierarchical differentiation competence in response to retinoic acid ensures stem cell maintenance during mouse spermatogenesis
Source: Development. 2015 May 1;142(9):1582–92. doi: 10.1242/dev.118695 (PMC4419276; doi:10.1242/dev.118695)
Supplement: Supplementary Material [file supp_dev.118695_DEV118695supp.pdf]

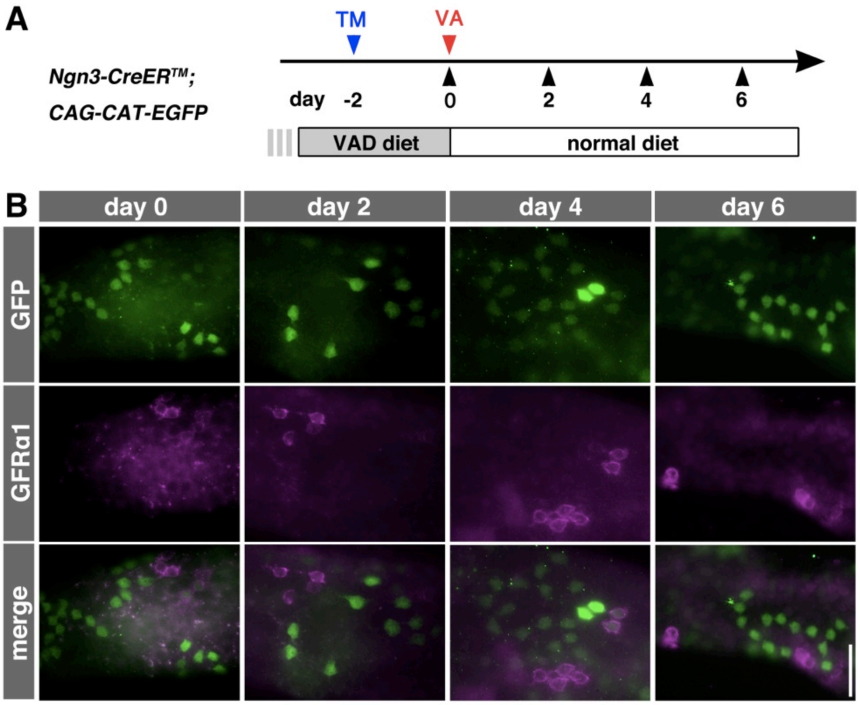

Fig. S1. Cell fate analyses of VAD model mice (related to Fig. 2). Fate of pulse-labeled NGN3+ cells following VA administration. The same protocol was used as in Fig. 2A. (A) The experimental schedule. (B) Representative images of the immunofluorescence (IF) of whole-mount seminiferous tubules 0, 2, 4, and 6 days after VA injection. GFP (green), GFRα1 (magenta). Scale bar = 50 μm.

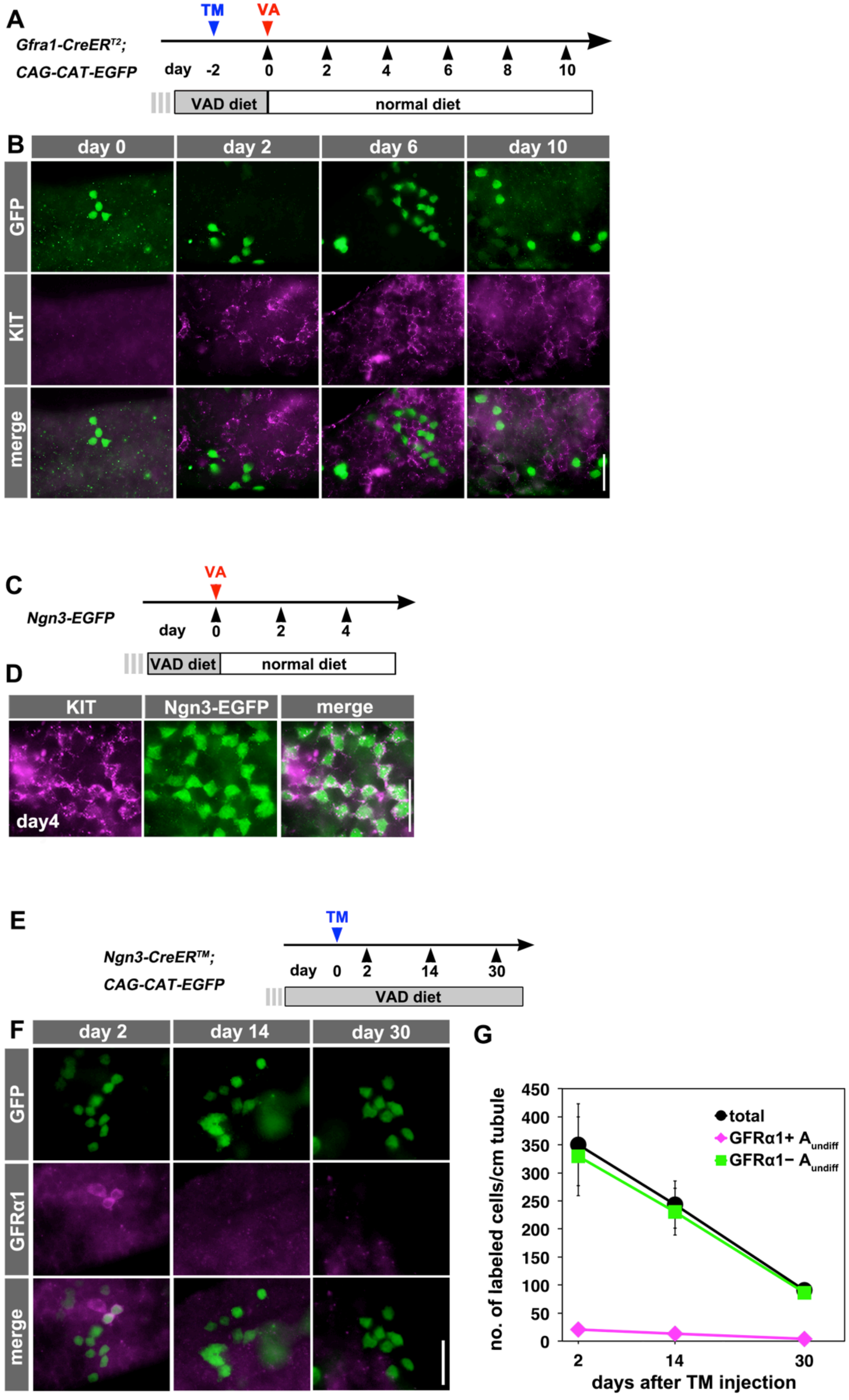
A
TM
VA
Gfra1-CreER^{T2};
CAG-CAT-EGFP
day -2 0 2 4 6 8 10
VAD diet
normal diet
B
day 0
day 2
day 6
day 10
GFP
KIT
merge
C
VA
Ngn3-EGFP
day 0 2 4
VAD diet
normal diet
D
KIT
Ngn3-EGFP
merge
day4
E
TM
Ngn3-CreER^{TM};
CAG-CAT-EGFP
day 0 2 14 30
VAD diet
F
day 2
day 14
day 30
GFP
GFRα1
merge
G
no. of labeled cells/cm tubule
days after TM injection
total
GFRα1+ A_{undiff}
GFRα1− A_{undiff}
450
400
350
300
250
200
150
100
50
0
2
14
30

Fig. S2. Cell fate analyses of VAD models (Related to Fig. 3). (A and B) Fate of pulse-labeled GFRα1+ cells following VA administration, performed in the same experiment shown in Fig. 3A–C. (A) The experimental schedule. (B) IF analysis of GFP (green) and KIT (magenta) expression in whole-mount seminiferous tubules 0, 2, 6, and 10 days after VA injection. (C and D) The origin of KIT+ spermatogonia in *Ngn3-EGFP* mice. In these mice, NGN3+ cells can be traced short-term according to half-life of the residual GFP of approximately 2–3 days. (C) The experimental schedule. Before and after VA injection, the testes samples were harvested at the indicated times. (D) IF for KIT (magenta) and GFP (green) in whole-mount seminiferous tubule of *Ngn3-EGFP* mouse 4 days after VA administration. Virtually all of the KIT+ cells were positive for residual GFP (mean ± s.e.m. values for three animals were 99.0 ± 0.2% and 99.2 ± 0.3% on days 2 and 4, respectively), indicating that essentially all KIT+ cells were derived from NGN3+ cells.
(E to G) Fate of pulse-labeled NGN3+ spermatogonia in *Ngn3-CreER^{TM}; CAG-CAT-EGFP* mice under conditions of persistent VAD. (E) The experimental schedule for (F) and (G). After a 4-hydroxytamoxifen (TM) pulse, *Ngn3-CreER^{TM}; CAG-CAT-EGFP* mice were continually fed the VAD diet, and the testes samples were harvested at the indicated time points. (F) IF images of whole-mount seminiferous tubule at 2, 14, and 30 days after incubation with antibodies against GFP (green) and GFRα1 (magenta). (G) Number of labeled GFRα1+ A_{undiff} (magenta), GFRα1− A_{undiff} (green), and total (black) cells in testes of *Ngn3-CreER^{TM}; CAG-CAT-EGFP* mice following the schedule in (E). Mean ± s.e.m. values for five and three mice on days 0 and 14, respectively, and the mean for two mice on day 30. Scale bars = 50 μm.

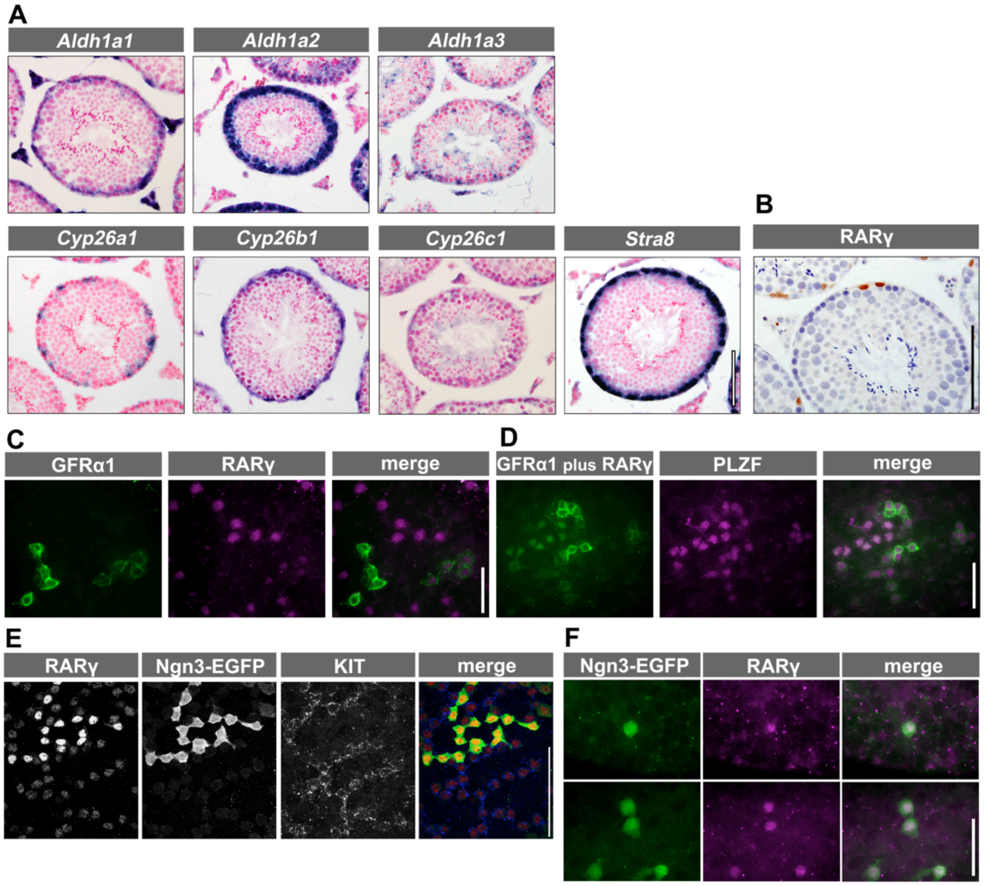

Fig. S3. Expression of RA-related genes (related to Fig. 4). (A) *In situ* hybridization of *Aldh1a1*, *Aldh1a2*, *Aldh1a3* (encoding enzymes synthesizing RA), *Cyp26a1, Cyp26b1, Cyp26c1* (encoding enzymes inactivating RA)*, and Stra8* (an RA target gene) in normal testis. *Aldh1a1* is expressed at intermediate levels by Sertoli cells and peritubular cells as well as by interstitial cells. *Aldh1a2* is expressed at high levels by pachytene spermatocytes. *Cyp26a1* is expressed by Sertoli cells, and *Cyp26b1* is expressed by peritubular myoid cells. Expression of these genes does not show any particular spatial pattern related to vasculature or interstitial cells, and expression is observed mainly surrounding the tubule circumference. Further, the finding *Stra8* evenly surrounded the seminiferous tubule circumference does not support the conclusion that RA signaling is distributed unevenly. Signals emitted by *Aldh1a3* and *Cyp26c1* were too faint to identify the expression pattern. Nuclei were stained with Nuclear Fast Red. These data are largely consistent with those of previous studies (Sugimoto et al., 2012; Vernet et al., 2006). (B) Representative images of a cross-section of

the testis stained with an antibody against RARγ (diaminobenzidine (DAB), brown; hematoxylin, blue). Expression of RARγ was observed only in spermatogonia. (C) Detection of GFRα1 (green) and RARγ (magenta) in whole mounts. Double-positive cells showed weaker signals for both proteins. (D) Whole-mount IF using a mixture of antibodies against RARγ and GFRα1 (green) and an antibody against PLZF (magenta). Virtually all PLZF+ cells expressed RARγ, GFRα1, or both. (E) Whole-mount IF of a seminiferous tubule of an *Ngn3-EGFP* mouse triple-stained with antibodies against RARγ (red), GFP (green), and KIT (blue). In addition to the strong RARγ signal detected in GFP+ cells, the image shows weaker staining of KIT+ cells. (F) Representative IF images of whole-mount seminiferous tubules from *Ngn3-EGFP* mice reacted with antibodies against GFP and RARγ. RARγ-expressing NGN3+ A_s and A_{pr} are shown. Scale bars = 100 μm (A,B,E) and 50 μm (C,D,F).

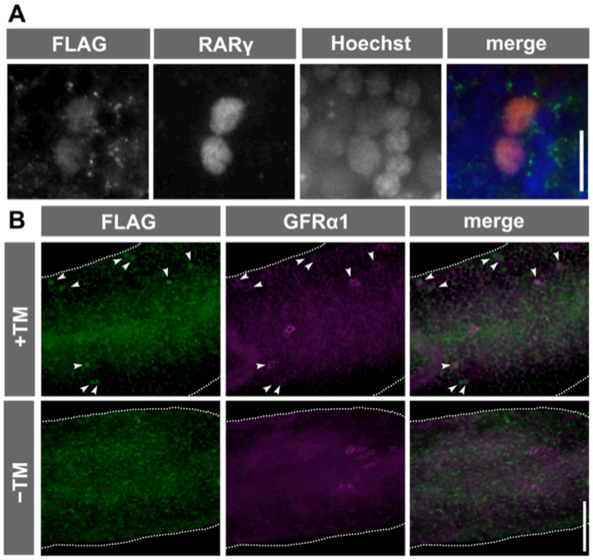

C

	day 2	day 30	day 50
total GFRα1+ cells	2451	873	2021
FLAG+/GFRα1+ cells	400	35	12
FLAG+ cells/total GFRα1+ cells (%)	16	4	0.6
observed total tubule length (cm)	7.68	2.77	6.13
GFRα1+ cells/cm tubule	319	315	329

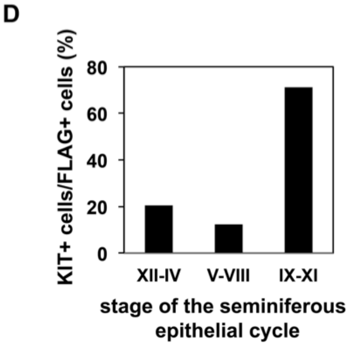

Fig. S4. Expression of FLAG-RARγ in *Gfra1-CreER^{T2};CAG-CAT-3xFLAG-Rarg* mice and the fate of FLAG-RARγ+ cells (related to Fig. 5). Whole-mount IF of seminiferous tubules of *Gfra1-CreER^{T2};CAG-CAT-3xFLAG-Rarg* mice. (A) Representative IF images of FLAG (green), RARγ (red), and Hoechst (blue) of a seminiferous tubule of an animal injected with TM 2 days earlier. (B) IF images for FLAG (green) and GFRα1 (magenta) of seminiferous tubules of mice injected with TM 2 days before (upper panels), or control animals not injected with TM (lower panels). Arrowheads indicate FLAG+ cells. (C) The contribution of FLAG-RARγ+ cells to the total GFRα1+ cell population, 2, 30 and 50 days after induction of FLAG-RARγ by TM injection. The total counts of cells from the indicated lengths of seminiferous tubules are summarized. Total counts of two testes on days 2 and one testis on days 30 and 50 are indicated. (D) The percentages of KIT+ cells among FLAG-RARγ + cells found in different seminiferous epithelial stages 4 days after TM injection. The figure shows 161, 57, and 76 FLAG+ cells that were analyzed for stages XII–IV, V–VIII, and IX–XI, respectively, in whole-mount seminiferous tubule specimens from a testis immunostained for FLAG and KIT. Staging of the seminiferous epithelium was performed according to published methods (Parvinen and Hecht, 1981). The stage was earlier by approximately three to four stages when FLAG-RARγ was induced, considering the lag between the TM injection and protein synthesis induction (1~2 days). Scale bars = 20 μm (A) and 100 μm (B).

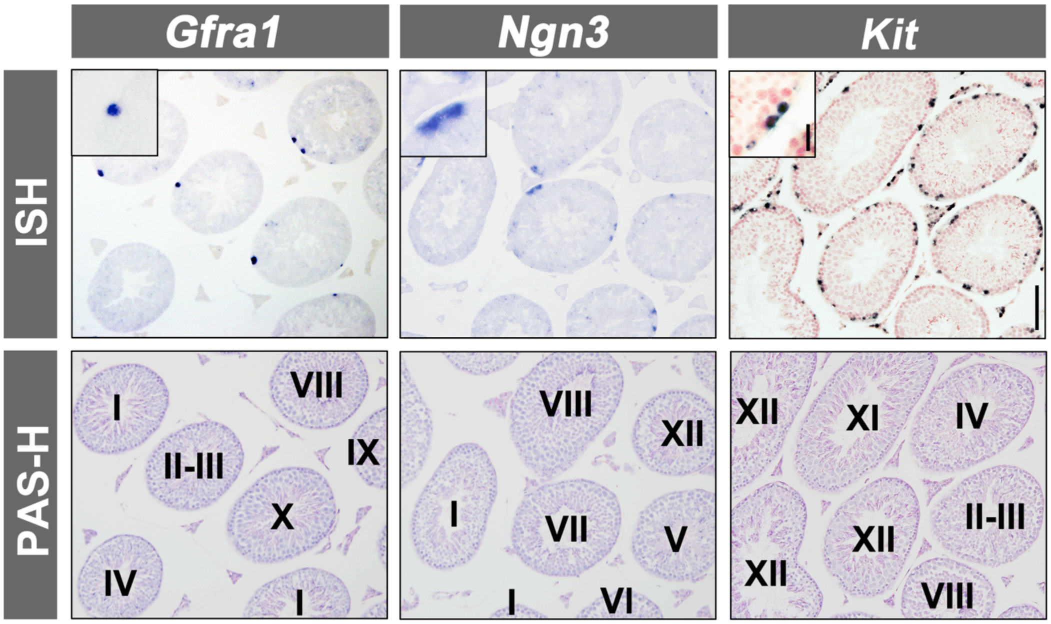

Fig. S5. Expression of *Gfra1* and *Ngn3* in the testes (related to Fig. 6). Images of *in situ* hybridization analysis of *Gfra1*, *Ngn3,* and *Kit* (top panels) and PAS-H staining (bottom panels) on adjacent sections of the undisturbed, wild-type mouse testis. Roman numerals indicate the stage of the seminiferous epithelial cycle. Nuclei were stained with Nuclear Fast Red only in the images of *in situ* hybridization analysis of *Kit*. Scale bar = 100 µm. Insets show high-magnification images. Scale bar = 20 µm.

Supplementary Materials and Methods

Generation of *CAG-CAT-3xFLAG-Rarg* transgenic mice

A *Bam*HI–*Xba*I fragment containing the *Rarg* coding sequence from a FANTOM cDNA clone G370007N15 (DNAFORM) and a fragment encoding a 3xFLAG-tag from pCMV-3Tag-6 (Agilent Technologies) were inserted into the plasmid pCAG-CAT (Kawamoto et al., 2000) to generate p*CAG-CAT-3xFLAG-Rarg*. A fragment containing the *CAG-CAT-3xFLAG-Rarg* region was microinjected into the pronuclei of single-cell embryos of C57BL/6J×C57BL/6J mice to produce transgenic mice (Laboratory Animal Resource Center, University of Tsukuba) in accordance with the University of Tsukuba's Guide for the Care and Use of Laboratory Animals with the approval of its Institutional Review Board.

Pulse labeling and induction of FLAG-RARγ by TM

Induction of Cre-mediated recombination by TM to label GFRα1+ and NGN3+ cells with GFP or to induce the FLAG-RARγ expression was performed as described previously (Hara et al., 2014; Nakagawa et al., 2010). Mice were injected i.p. with the indicated dose of TM (Sigma-Aldrich) per individual. TM was dissolved in DMSO, ethanol, and then sesame oil (Nacalai Tesque). The mouse strains, which were injected when they were 10–17 weeks of age, were as follows: 1. Subjected to VAD: *Ngn3-CreER*^{TM}*; CAG-CAT-EGFP* (0.5 mg), *Gfra1-CreER*^{T2}*; CAG-CAT-EGFP* (1.0 mg), and *Gfra1-CreER*^{T2}*; CAG-CAT-3xFLAG-Rarg* (1.0 mg); and 2. Maintained under normal conditions: *Gfra1-CreER*^{T2}*; CAG-CAT-EGFP* (2.0 mg) and *Gfra1-CreER*^{T2}*; CAG-CAT-3xFLAG-Rarg* (2.0 mg).

In situ hybridization

Under deep anesthesia induced using avertin, mice were perfused with 4% paraformaldehyde (PFA) in phosphate-buffered saline (PBS), and the testes were excised, embedded in paraffin, and sectioned. The sections were hybridized with digoxigenin-labeled riboprobes, and the hybrids were detected using BM purple AP substrate (Roche) according to a published method (Yoshida et al., 2001). *Ngn3*, *Kit*, *Aldh1a1, Aldh1a2, Aldh1a3, Cyp26b1, Cyp26c1 and Stra8* probes were described previously (Sugimoto et al., 2012; Yoshida et al., 2004), and the IMAGE EST clone #6390018,

Accession #BC054378 (Invitrogen, Life Technologies) was used to prepare a *Gfra1* probe. Stages of the seminiferous epithelium were judged from the adjacent section stained with periodic acid-Schiff (PAS) hematoxylin.

IF analysis

Whole-mount IF of seminiferous tubules was performed according to a published method (Nakagawa et al., 2010). After the testes were dissected in PBS, untangled seminiferous tubules were fixed for 3 hours in 4% PFA in PBS and attached to an MAS-coated glass slide (Matsunami Glass). The samples were then dehydrated using a methanol series and rehydrated in TBST (TBS containing 0.1% Tween 20). After the samples were washed twice for 10 minutes each in TBST, they were saturated for 1 hour in TNB blocking buffer (PerkinElmer) containing 4% donkey serum (Jackson ImmunoResearch) and 0.01% Hoechst 33342 (Invitrogen, Life Technologies). Samples were next incubated at 4°C for 12 hours with primary antibody in TNB blocking buffer containing donkey serum at the dilutions indicated below and washed three times in TBST for 15 minutes each. Tissues were then incubated with secondary antibody for 1 hour at room temperature. The observations and measurements were performed using a BX51 upright fluorescence microscope equipped with a DP72 CCD camera (Olympus) or using a Leica TCS SP8 Confocal System. Primary antibodies used in this study and their dilution factors (in parentheses) were as follows: anti-GFRα1 goat polyclonal antibody (R&D Systems, #AF560, 1:200); anti-GFP rabbit, rat, or chicken polyclonal antibodies (Invitrogen, Life Technologies, #A11122, 1:300; Nacalai Tesque, #04404-84, 1:200; or Abcam, #13970, 1:200, respectively); anti-KIT rat monoclonal antibody (BD Biosciences, #553352, 1:200); anti-RARγ1 rabbit monoclonal antibody (Cell Signaling Technologies, #8965, 1:200); anti-DDDDK rabbit polyclonal antibody (MBL, #PM020, 1:500); anti-FLAG mouse monoclonal antibody (Sigma, #F1804, 1:500); and anti-PLZF mouse monoclonal antibody (Calbiochem, #OP128, 1:50). The secondary antibodies manufactured at Invitrogen, Life Technologies were as follows (all diluted 1:400): Alexa 488-conjugated anti-goat (#A11055), anti-rabbit (#A21206), anti-rat (#A21208) IgGs; Alexa 594-conjugated anti-goat (#A11058), anti-rabbit (#A21207), anti-rat (#A21209) IgGs; and Alexa 647-conjugated anti-goat (#A21447) IgG.

Immunohistochemistry

For immunostaining paraffin sections, testes were fixed in 4% PFA–PBS for 12 hours at 4°C, dehydrated, and then embedded in paraffin. Deparaffinized sections were blocked with blocking reagent (PerkinElmer) and incubated with an anti-RARγ1 rabbit monoclonal antibody (1:800). The immunoreaction was visualized using a biotin-conjugated anti-rabbit secondary antibody (Vector labs) in combination with an ABC Kit (Vector labs) and a DAB substrate Kit (Vector labs). Finally, the sections were stained with hematoxylin (Wako) to visualize nuclei.

Microarray analysis of gene expression

Total RNA was extracted using the RNeasy Micro Kit (Qiagen). RNA was converted to cDNA, and cRNA was then amplified using the Low Input Quick Amp Labeling Kit (Agilent Technologies). Cyanine 3-CTP-labeled cRNAs were combined and hybridized to the SurePrint G3 Mouse GE 8x60K Microarray (Agilent Technologies) using a Gene Expression Hybridization Kit (Agilent Technologies). After hybridization, arrays were washed and dried according to the manufacturer's instructions. Arrays were scanned using an Agilent Technologies Scanner (G2505C and G2565CA) with the default settings for 8 × 60k-format one-color arrays. Images were analyzed using Feature Extraction ver.10.7.3.1 (Agilent Technologies). Three samples from different animals were used to analyze each spermatogonial fraction (for NGN3+ cells, samples from two mice were pooled). Data preparation and statistical analysis were performed using Gene Spring v12.0.0.0 (Silicon Genetics). Data correction was performed with the threshold raw signals set to 1.0, percent shift to the 75th percentile as normalization algorithm, and no baseline transformation. Genes selected for display in Fig. 4A and Table S2 are associated with the following gene ontogeny (GO) terms: retinoic acid receptor activity, retinoic acid receptor binding, retinoic acid receptor signaling pathway, regulation of retinoic acid receptor signaling pathway, retinoic acid 4-hydroxylase histone acetyltransferase complex, mediator complex, histone deacetylase complex, ligand-dependent chromatin remodeling, and ATP-dependent chromatin remodeling.

Quantitative RT-PCR

Total RNA was isolated using an mirVana miRNA Isolation Kit (Ambion, Life Technologies) and reverse-transcribed using SuperScript III First-Strand Synthesis SuperMix for qRT-PCR primed

with a mixture of oligo(dT) and random hexamers (Invitrogen, Life Technologies). After treatment with DNase I (Ambion, Life Technologies), quantitative PCR was performed using TaqMan Universal PCR Master Mix and probes for *Rarg* (Mm00441091_m1), *Rara* (Mm01296312_m1), and *Actb* (Mm00607939_s1) (Applied Biosystems, Life Technologies) using a LightCycler 480 system (Roche).

Supplementary Reference

Parvinen, M. and Hecht, N. B. (1981). Identification of living spermatogenic cells of the mouse by transillumination-phase contrast microscopic technique for 'in situ' analyses of DNA polymerase activities. *Histochemistry* **71**, 567-579.

Table S1. Numbers of spermatogonia versus Sertoli cells in VAD mouse testes (related to Fig. 1F)

**Days after VA readministration**	***Gfra1+* spermatogonia**	**Sertoli cells**	***Ngn3+* spermatogonia**	**Sertoli cells**	***Kit+* spermatogonia**	**Sertoli cells**
**day 0**	378	12349	2401	15757	0	7566
**day 2**	181	6641	308	7455	1651	7286
**day 4**	367	8247	114	9593	3226	8947

Total counts of spermatogonia and Sertoli cells in sections prepared from two or three testes of VAD mice probed with *Gfra1, Ngn3*, and *Kit*. Counts are summarized in Fig. 1G.

Table S2. Microarray analysis of retinoic acid-related gene expression by GFRα1+ and NGN3+ cells

Ratio are represented as log2([Ngn3+]/[GFRα1+]), where [Ngn3+] and [GFRα1+] indicate the average signal intensities obtained from Ngn3+ and GFRα1+ spermatogonia, respectively (n = 3).
P values are calculated usingt test with Benjamini-Hochberg correction for false discovery rate.

ProbeName	GeneSymbol	EntrezGeneID	ratio	p (Corr)
A_51_P474658	Esr1	13982	2.28	0.006
A_55_P2051716	Tbx1	21380	1.87	0.015
A_52_P237077	Esr1	13982	1.64	0.006
A_55_P2151056	Rarg	19411	1.64	0.007
A_51_P501844	Cyp26b1	232174	1.62	0.006
A_51_P226711	Ptf1a	19213	1.61	0.102
A_55_P2164040	Tada2b	231151	1.34	0.022
A_55_P2065074	Hdac9	79221	1.32	0.103
A_55_P2019009	Ncoa7	211329	1.24	0.081
A_55_P2036620	Lrif1	321000	1.17	0.074
A_66_P114585	Mta3	116871	1.11	0.016
A_55_P2030883	Esr1	13982	0.94	0.041
A_55_P2027822	Ccdc101	75565	0.91	0.006
A_51_P140797	Dcaf6	74106	0.89	0.014
A_55_P1977850	Sall1	58198	0.87	0.023
A_51_P152747	Nrip1	268903	0.82	0.018
A_52_P536927	Satb2	212712	0.81	0.162
A_51_P399845	Fgf2	14173	0.78	0.061
A_55_P2018559	Morf4l1	21761	0.78	0.037
A_55_P1981794	Ccdc101	75565	0.74	0.007
A_55_P2369474	Nr1h4	20186	0.71	0.263
A_55_P2128597	Nr1h3	22259	0.68	0.036
A_55_P1993168	Ppargc1b	170826	0.65	0.275
A_66_P120249	Nr1h3	22259	0.64	0.021
A_66_P126138	Ncoa1	17977	0.60	0.043

A_55_P2061386	Chd3	216848	0.56	0.030
A_52_P413584	Nrip1	268903	0.55	0.427
A_52_P656545	Ogt	108155	0.52	0.068
A_55_P2163438	Actn2	11472	0.50	0.071
A_55_P2134616	Med12l	329650	0.50	0.215
A_55_P2025463	Brpf3	268936	0.48	0.073
A_55_P2130975	Ncoa1	17977	0.44	0.102
A_55_P2031367	Brd1	223770	0.39	0.077
A_52_P334562	Vdr	22337	0.39	0.280
A_51_P106799	Pparg	19016	0.37	0.043
A_51_P108266	Actn2	11472	0.36	0.413
A_55_P1983006	Hdac5	15184	0.35	0.171
A_51_P441387	Kansl1l	68691	0.33	0.237
A_51_P307370	Rxra	20181	0.32	0.119
A_55_P1958532	Hr	15460	0.32	0.301
A_52_P494686	Kansl1l	68691	0.32	0.068
A_55_P2244722	Taf9b	407786	0.31	0.006
A_52_P58145	Aldh1a2	19378	0.28	0.036
A_55_P2095342	Rara	19401	0.23	0.084
A_55_P2153461	Cecr2	330409	0.21	0.149
A_55_P2082070	Taf9	108143	0.21	0.179
A_55_P1986993	Brd8	78656	0.20	0.541
A_55_P2135383	Taf6l	225895	0.19	0.318
A_55_P1978481	Nr1h2	22260	0.18	0.325
A_55_P2415372	Mta1	116870	0.17	0.180
A_55_P2080542	Rbbp7	245688	0.16	0.361
A_55_P2036615	Lrif1	321000	0.13	0.220
A_66_P130582	Ncoa1	17977	0.12	0.583
A_55_P1967618	Med16	216154	0.12	0.262

A_51_P279038	Ppargc1a	19017	0.12	0.717
A_51_P481191	Hdac8	70315	0.11	0.683
A_51_P508853	Ep400	75560	0.11	0.901
A_51_P213334	Hdac11	232232	0.11	0.522
A_66_P129029	Taf10	24075	0.09	0.452
A_66_P120260	Taf10	24075	0.09	0.464
A_51_P432199	Sap30	60406	0.09	0.560
A_55_P2024654	Trrap	100683	0.08	0.616
A_65_P17492	Med29	67224	0.08	0.509
A_52_P175952	Map3k7	26409	0.06	0.698
A_51_P417839	Hdac4	208727	0.05	0.732
A_55_P2185548	Cdk8	264064	0.04	0.785
A_55_P2022094	Mta3	116871	0.03	0.832
A_55_P2162537	Ing4	28019	0.03	0.884
A_55_P1960873	Mbd3	17192	0.03	0.722
A_66_P127024	Mbd3	17192	0.02	0.876
A_55_P2008609	Rxrb	20182	0.02	0.832
A_55_P1993820	Kdm1a	99982	0.02	0.860
A_51_P141521	Sap130	269003	0.01	0.944
A_52_P589550	Nr2c1	22025	0.01	0.983
A_52_P319541	Med12l	329650	0.00	0.999
A_55_P2077608	Hdac1	433759	0.00	0.999
A_51_P350503	Csrp2bp	228714	-0.01	0.992
A_66_P138072	Esr1	13982	-0.01	0.984
A_52_P464062	Hdac8	70315	-0.01	0.978
A_55_P2170076	Taf9	108143	-0.02	0.893
A_51_P392861	Med25	75613	-0.02	0.938
A_55_P2036894	Med29	67224	-0.03	0.652
A_55_P2084807	Rarb	218772	-0.04	0.969

A_55_P1960626	Satb2	212712	-0.05	0.969
A_55_P2049086	Map3k7	26409	-0.05	0.717
A_52_P325477	Trim16	94092	-0.05	0.826
A_65_P01783	Med28	66999	-0.07	0.589
A_55_P1992655	Hdac6	15185	-0.07	0.458
A_66_P128384	Hdac1	433759	-0.08	0.678
A_55_P1957028	Kat7	217127	-0.08	0.614
A_51_P103757	Kat8	67773	-0.08	0.372
A_66_P124715	Ppard	19015	-0.08	0.717
A_52_P238044	Psmc3ip	19183	-0.08	0.718
A_55_P2171993	Mrgbp	73247	-0.08	0.467
A_55_P2072551	Ncoa6	56406	-0.09	0.917
A_55_P2025974	Ruvbl2	20174	-0.09	0.368
A_52_P579876	Suds3	71954	-0.10	0.646
A_55_P2154690	Smarca4	20586	-0.11	0.653
A_55_P1971347	Mta3	116871	-0.11	0.476
A_55_P2081560	Phf20	228829	-0.11	0.547
A_55_P2087825	Ncoa2	17978	-0.11	0.336
A_52_P561927	Ncor1	20185	-0.12	0.467
A_52_P376106	Slc30a9	109108	-0.13	0.450
A_52_P402319	Med20	56771	-0.13	0.298
A_51_P492087	Pole3	59001	-0.13	0.280
A_55_P1962154	Rarb	218772	-0.14	0.803
A_55_P2080794	Taf9	108143	-0.14	0.343
A_51_P465772	Ruvbl1	56505	-0.15	0.329
A_66_P102232	Hmga1	15361	-0.15	0.795
A_55_P2095345	Rara	19401	-0.15	0.473
A_52_P370473	Msl1	74026	-0.16	0.211
A_66_P100789	Kansl2	69612	-0.16	0.228

A_55_P1956712	Med28	66999	-0.16	0.043
A_51_P416419	Calr	12317	-0.17	0.061
A_51_P199567	Med26	70625	-0.18	0.064
A_55_P2036459	Med24	23989	-0.18	0.286
A_52_P413623	Usp22	216825	-0.18	0.107
A_55_P2003789	Taf4a	228980	-0.18	0.150
A_55_P2025293	Ss18	268996	-0.18	0.177
A_55_P1987302	Eny2	223527	-0.18	0.083
A_51_P349662	Taf5l	102162	-0.19	0.246
A_51_P483118	Hmga1	15361	-0.20	0.208
A_55_P1987449	Ep400	75560	-0.20	0.102
A_55_P1955722	Med15	94112	-0.20	0.096
A_55_P2128263	Phf21a	192285	-0.20	0.383
A_55_P2154684	Smarca4	20586	-0.21	0.142
A_55_P2147591	Med27	68975	-0.21	0.030
A_51_P161874	Med30	69790	-0.21	0.083
A_55_P1964033	Carm1	59035	-0.21	0.184
A_66_P107585	Taf2	319944	-0.22	0.247
A_55_P2009792	Hdac6	15185	-0.23	0.030
A_55_P1970775	Hdac1	433759	-0.23	0.083
A_55_P1962419	Ccar1	67500	-0.23	0.084
A_55_P2036526	Mbd2	17191	-0.23	0.346
A_55_P2016877	Nsd1	18193	-0.23	0.159
A_51_P159415	Sin3a	20466	-0.24	0.078
A_66_P111773	Gatad2a	234366	-0.26	0.133
A_55_P2099585	Rarb	218772	-0.26	0.722
A_51_P164207	Mta2	23942	-0.26	0.123
A_51_P116027	Med27	68975	-0.27	0.023
A_55_P2092501	Med1	19014	-0.27	0.116

A_55_P2003517	Zfp536	243937	-0.27	0.259
A_55_P2123137	Ncor2	20602	-0.27	0.146
A_51_P226053	Sra1	24068	-0.27	0.036
A_55_P2049602	Kat2a	14534	-0.28	0.043
A_55_P2107120	Kansl2	69612	-0.28	0.071
A_55_P2165969	Ncor1	20185	-0.28	0.331
A_51_P496400	Med9	192191	-0.29	0.060
A_55_P2051727	Rnf8	58230	-0.29	0.020
A_55_P1972653	Tada3	101206	-0.29	0.010
A_55_P1996742	Rbm14	56275	-0.29	0.318
A_55_P1987504	Ccnc	51813	-0.29	0.159
A_55_P2123822	Rbbp7	245688	-0.29	0.053
A_55_P2018407	Sycp3	20962	-0.30	0.014
A_51_P116007	Hdac2	15182	-0.30	0.030
A_55_P2054540	Crebbp	12914	-0.30	0.292
A_55_P2076057	Hmga1	15361	-0.31	0.060
A_55_P2108334	Med10	28077	-0.31	0.180
A_51_P218953	Zfp536	243937	-0.31	0.383
A_51_P141554	Med22	20933	-0.31	0.065
A_55_P1975640	Zfp217	228913	-0.31	0.118
A_55_P2057291	Kansl3	226976	-0.32	0.018
A_52_P219943	Epc1	13831	-0.32	0.171
A_55_P1972852	Mcrs1	51812	-0.32	0.048
A_51_P396708	Med21	108098	-0.32	0.020
A_55_P2125241	Brd1	223770	-0.33	0.180
A_51_P286814	Ncor2	20602	-0.33	0.116
A_55_P2165974	Ncor1	20185	-0.33	0.179
A_65_P19089	Esrrg	26381	-0.33	0.476
A_51_P202014	Taf12	66464	-0.33	0.137

A_55_P2072233	Zcchc12	72693	-0.33	0.176
A_51_P349803	Med11	66172	-0.34	0.150
A_51_P373369	Med4	67381	-0.34	0.087
A_51_P307325	Rbbp4	19646	-0.34	0.136
A_51_P261107	Ogt	108155	-0.34	0.103
A_51_P432069	Trrap	100683	-0.34	0.516
A_52_P504068	Cdk8	264064	-0.34	0.007
A_52_P78875	Phf12	268448	-0.35	0.142
A_51_P380986	Epc1	13831	-0.35	0.054
A_51_P172842	Med19	381379	-0.35	0.048
A_52_P231737	Actl6a	56456	-0.35	0.029
A_55_P2083654	Prmt2	15468	-0.35	0.171
A_55_P2048202	Ss18	268996	-0.36	0.058
A_55_P1993109	Taf6	21343	-0.36	0.030
A_52_P497056	Supt20	56790	-0.36	0.147
A_55_P2111419	Eny2	223527	-0.36	0.048
A_51_P370286	Med24	23989	-0.37	0.030
A_52_P197666	Med12	59024	-0.37	0.012
A_55_P2044359	Rxrg	20183	-0.38	0.331
A_55_P2071868	Ncoa3	17979	-0.38	0.068
A_55_P2034400	Cdk8	264064	-0.38	0.014
A_52_P529486	Wdr77	70465	-0.38	0.016
A_66_P132855	Baz1b	22385	-0.38	0.079
A_51_P212630	Chd8	67772	-0.38	0.063
A_55_P1965629	Hcfc1	15161	-0.39	0.008
A_55_P1959953	Helz2	229003	-0.39	0.272
A_55_P2128270	Phf21a	192285	-0.39	0.122
A_51_P116616	Rbm14	56275	-0.39	0.030
A_51_P510663	Supt3	109115	-0.40	0.060

A_55_P2077879	Kat6b	54169	-0.40	0.052
A_55_P1955676	Csrp2bp	228714	-0.40	0.024
A_55_P2132502	Pkn1	320795	-0.40	0.012
A_55_P1974412	Kat2a	14534	-0.40	0.041
A_52_P391018	Phf16	382207	-0.41	0.201
A_66_P119017	Smarca1	93761	-0.41	0.378
A_66_P133043	Mbd2	17191	-0.41	0.079
A_55_P2019004	Ncoa7	211329	-0.41	0.603
A_52_P110291	Phf12	268448	-0.42	0.015
A_52_P117197	Epc1	13831	-0.42	0.007
A_55_P1996329	Kat5	81601	-0.43	0.009
A_55_P2033521	Med6	69792	-0.43	0.046
A_55_P2002314	Zmiz2	52915	-0.43	0.037
A_55_P2074331	Rxra	20181	-0.43	0.126
A_55_P1991802	Csrp2bp	228714	-0.43	0.025
A_55_P2139753	Phf15	76901	-0.43	0.043
A_52_P101333	Sap18	20220	-0.44	0.121
A_55_P2153459	Cecr2	330409	-0.44	0.030
A_51_P423880	Smarcd3	66993	-0.44	0.096
A_55_P2017714	Kat5	81601	-0.44	0.033
A_55_P1979356	Morf4l1	21761	-0.44	0.018
A_55_P2138120	Atxn7l3	217218	-0.44	0.042
A_51_P263220	Taf5	226182	-0.45	0.024
A_55_P2016712	Msl1	74026	-0.45	0.030
A_51_P513311	Rxrg	20183	-0.45	0.324
A_52_P538709	Tada3	101206	-0.46	0.008
A_55_P1973402	Tada2a	217031	-0.46	0.006
A_55_P2077884	Kat6b	54169	-0.46	0.152
A_55_P1964009	Zfp217	228913	-0.46	0.063

A_55_P2098688	Mcrs1	51812	-0.46	0.041
A_51_P100787	Snw1	66354	-0.47	0.044
A_51_P141104	Med18	67219	-0.47	0.020
A_52_P266459	Ing2	69260	-0.47	0.101
A_51_P111455	Wdr77	70465	-0.47	0.014
A_55_P2054240	Med8	80509	-0.48	0.009
A_51_P397768	Csnk2a1	12995	-0.48	0.017
A_52_P176300	Med1	19014	-0.48	0.007
A_51_P233788	Dmap1	66233	-0.48	0.025
A_55_P2108324	Med12	59024	-0.49	0.090
A_51_P465809	Slc30a9	109108	-0.49	0.037
A_55_P1967617	Med16	216154	-0.49	0.176
A_55_P1968464	Kat6a	244349	-0.49	0.044
A_55_P2078776	Mll5	69188	-0.49	0.016
A_55_P1992640	Hdac3	15183	-0.50	0.015
A_51_P129149	Gatad2a	234366	-0.51	0.038
A_55_P2004385	Csrp2bp	228714	-0.51	0.015
A_51_P463828	Baz1b	22385	-0.51	0.110
A_55_P1958306	Thrap3	230753	-0.51	0.023
A_52_P653902	Eny2	223527	-0.52	0.090
A_55_P2146404	Kansl1	76719	-0.52	0.030
A_52_P3412	Med17	234959	-0.52	0.031
A_55_P2095365	Meaf6	70088	-0.53	0.023
A_55_P2111875	Taf12	66464	-0.53	0.371
A_55_P1994917	Wdr5	140858	-0.53	0.016
A_55_P2134356	Msl3	17692	-0.53	0.030
A_55_P1963606	Hcfc1	15161	-0.55	0.095
A_52_P282987	Yeats2	208146	-0.55	0.021
A_55_P1971828	Csnk2a1	12995	-0.56	0.007

A_51_P203474	Tsg101	22088	-0.56	0.030
A_52_P647291	Cecr2	330409	-0.57	0.058
A_55_P2116853	Brpf1	78783	-0.57	0.007
A_55_P2083814	Zzz3	108946	-0.57	0.006
A_51_P274465	Sycp3	20962	-0.57	0.050
A_51_P265338	Nr0b2	23957	-0.58	0.480
A_55_P2056100	Pus1	56361	-0.58	0.009
A_55_P2121466	Ncor1	20185	-0.58	0.104
A_55_P2016708	Msl1	74026	-0.59	0.007
A_51_P255387	Med31	67279	-0.59	0.006
A_55_P2026889	Med10	28077	-0.59	0.007
A_55_P2070179	Ing4	28019	-0.59	0.029
A_66_P112546	Yeats4	64050	-0.60	0.015
A_55_P2092526	Tgif1	21815	-0.60	0.057
A_55_P2085664	Meaf6	70088	-0.60	0.006
A_55_P2112667	Fshr	14309	-0.60	0.179
A_66_P135192	Msl2	77853	-0.60	0.038
A_55_P2033520	Med6	69792	-0.60	0.006
A_51_P173384	Tada1	27878	-0.60	0.012
A_55_P2022845	Csnk2a1	12995	-0.60	0.006
A_55_P1975341	Nasp	50927	-0.60	0.045
A_52_P502267	Epc2	227867	-0.61	0.016
A_55_P1989858	Thrap3	230753	-0.61	0.009
A_55_P2236607	Ccnc	51813	-0.61	0.020
A_66_P129111	Nasp	50927	-0.62	0.107
A_55_P1968698	Rere	68703	-0.63	0.066
A_51_P481644	Mbip	217588	-0.63	0.032
A_52_P424767	Rbbp4	19646	-0.64	0.023
A_51_P324082	Med1	19014	-0.65	0.007

A_55_P2095727	Pml	18854	-0.65	0.015
A_52_P411601	Ep300	328572	-0.65	0.006
A_55_P2041397	Ezh2	14056	-0.66	0.028
A_52_P327467	Med7	66213	-0.67	0.009
A_55_P2112737	Actb	11461	-0.67	0.006
A_55_P2179834	Gatad2a	234366	-0.68	0.018
A_55_P1960281	Med7	66213	-0.70	0.007
A_55_P2024095	Med1	19014	-0.70	0.021
A_55_P2237432	Smarca1	93761	-0.71	0.103
A_52_P462350	Dr1	13486	-0.72	0.007
A_51_P255565	Smarcad1	13990	-0.73	0.018
A_55_P1965836	Crebbp	12914	-0.73	0.007
A_55_P1977593	Epc2	227867	-0.74	0.041
A_55_P2127804	Map3k7	26409	-0.75	0.064
A_52_P565940	Nsd1	18193	-0.75	0.005
A_52_P264229	Ing5	66262	-0.75	0.007
A_55_P2090179	Cbx5	12419	-0.76	0.025
A_52_P80593	Pole3	59001	-0.77	0.006
A_55_P1970755	Hdac9	79221	-0.79	0.101
A_55_P2033413	Med4	67381	-0.79	0.006
A_55_P2180551	Fam60a	56306	-0.80	0.008
A_52_P515826	Med13	327987	-0.80	0.130
A_55_P2033932	Zzz3	108946	-0.81	0.071
A_55_P2035407	Cyp26c1	546726	-0.81	0.014
A_55_P2090535	Thrap3	230753	-0.81	0.013
A_55_P1968200	Hjurp	381280	-0.81	0.048
A_55_P2018457	Hdac7	56233	-0.81	0.021
A_51_P224517	Phf17	269424	-0.82	0.006
A_55_P1971804	Hdac10	170787	-0.84	0.007

A_55_P2078770	Mll5	69188	-0.84	0.009
A_66_P127255	Ing3	71777	-0.88	0.007
A_55_P1986902	Med23	70208	-0.88	0.014
A_51_P136792	Calcoco1	67488	-0.90	0.014
A_51_P502764	Hdac10	170787	-0.90	0.006
A_51_P457358	Med13l	76199	-0.90	0.041
A_55_P2006499	Esrrg	26381	-0.91	0.065
A_65_P01247	Hjurp	381280	-0.91	0.014
A_55_P2011702	Ppargc1a	19017	-0.91	0.007
A_52_P382149	Cyp26a1	13082	-0.92	0.030
A_55_P2161267	Uimc1	20184	-0.95	0.177
A_55_P2048705	Fgf2	14173	-0.95	0.012
A_55_P2022840	Csnk2a1	12995	-0.96	0.014
A_55_P1998947	Trim16	94092	-0.97	0.069
A_55_P2072556	Ncoa6	56406	-0.99	0.020
A_65_P20174	Phf17	269424	-1.00	0.006
A_65_P07450	Brd8	78656	-1.01	0.041
A_65_P20249	Prkcb	18751	-1.01	0.024
A_55_P2045096	Hjurp	381280	-1.01	0.010
A_51_P257762	Kat2b	18519	-1.03	0.014
A_55_P2020373	Chd4	107932	-1.04	0.006
A_51_P450924	Pole4	66979	-1.06	0.048
A_66_P107680	Med8	80509	-1.06	0.006
A_55_P1989865	Thrap3	230753	-1.07	0.008
A_55_P2360661	Cbx5	12419	-1.11	0.003
A_55_P2183750	Ncoa3	17979	-1.12	0.006
A_55_P2075636	Epc2	227867	-1.18	0.020
A_52_P83959	Taf7	24074	-1.25	0.004
A_55_P2059134	Smarca5	93762	-1.25	0.006

A_55_P2062727	Nasp	50927	-1.25	0.006
A_52_P520940	Taf7	24074	-1.25	0.084
A_55_P2072443	Kansl1	76719	-1.25	0.084
A_55_P2078765	Mll5	69188	-1.27	0.008
A_52_P241544	Supt7l	72195	-1.28	0.023
A_66_P128997	Pml	18854	-1.29	0.003
A_55_P1981724	Nr2c1	22025	-1.31	0.012
A_55_P1994052	Asxl1	228790	-1.40	0.007
A_55_P2066329	Med28	66999	-1.41	0.326
A_52_P154880	Ing3	71777	-1.46	0.014
A_66_P119170	Phf21a	192285	-1.47	0.007
A_55_P1976278	Prkcb	18751	-1.58	0.006
A_55_P2163837	Med14	26896	-1.73	0.006
A_52_P406371	Med14	26896	-1.84	0.006
A_51_P137094	Sall2	50524	-1.95	0.060
A_66_P136955	Med14	26896	-2.06	0.005
A_52_P87843	Aldh1a3	56847	-4.73	0.004

Table S3. Counts of spermatogonia versus Sertoli cells during the seminiferous epithelial cycle

**stage**	***Gfra1+* spermatogonia (n = 7 testes)**	**Sertoli cells**	***Gfra1+* spermatogonia/ Sertoli cells**	***Ngn3+* spermatogonia (n = 5 testes)**	**Sertoli cells**	***Ngn3+* spermatogonia/ Sertoli cells**
I	105	2675	0.0393	47	3019	0.0156
II-III	60	1486	0.0404	54	1608	0.0336
IV	42	1597	0.0263	125	1737	0.0720
V	73	1905	0.0383	121	2238	0.0541
VI	34	1246	0.0273	116	1507	0.0770
VII	120	2612	0.0459	208	2749	0.0757
VIII	39	1153	0.0338	48	1086	0.0442
IX	63	1282	0.0491	58	1399	0.0415
X	36	891	0.0404	38	1006	0.0378
XI	59	1025	0.0576	33	1158	0.0285
XII	82	1592	0.0515	11	1736	0.0063

Total counts from seven (*Gfra1*) or five (*Ngn3*) testis sections probed with *Gfra1* and *Ngn3*.
